# BAF60/SWP73 subunits define subclasses of SWI/SNF chromatin remodelling complexes in Arabidopsis

**DOI:** 10.1111/nph.70182

**Published:** 2025-05-22

**Authors:** Sebastian P. Sacharowski, Szymon Kubala, Pawel Cwiek, Jaroslaw Steciuk, Dominika Gratkowska‐Zmuda, Paulina Oksinska, Ernest Bucior, Anna T. Rolicka, Monika Ciesla, Klaudia Nowicka, Saleh Alseekh, Takayuki Tohge, Patrick Giavalisco, Dorota L. Zugaj, Sara C. Stolze, Anne Harzen, Rainer Franzen, Bruno Huettel, Elzbieta Grzesiuk, Mohammad‐Reza Hajirezaei, Hirofumi Nakagami, Csaba Koncz, Alisdair R. Fernie, Tomasz J. Sarnowski

**Affiliations:** ^1^ Institute of Biochemistry and Biophysics Polish Academy of Sciences Pawinskiego 5A 02‐106 Warsaw Poland; ^2^ Max Planck Institute of Molecular Plant Physiology Am Mühlenberg 1 14476 Potsdam‐Golm Germany; ^3^ Center for Plant Systems Biology and Biotechnology 4000 Plovdiv Bulgaria; ^4^ Max Planck Institute for Plant Breeding Research Carl‐von‐Linné‐Weg 10 50829 Köln Germany; ^5^ Max Planck Genome Centre Cologne D‐50820 Köln Germany; ^6^ Leibniz Institute of Plant Genetics and Crop Plant Research Corrensstraße 3 D‐06466 Seeland OT Gatersleben Germany

**Keywords:** Arabidopsis, chromatin remodelling complexes, hormonal signalling, metabolome, SWI/SNF, SWP73/BAF60

## Abstract

- Evolutionarily conserved switch‐defective/sucrose nonfermentable (SWI/SNF) ATP‐dependent chromatin remodelling complexes (CRCs) alter nucleosome positioning and chromatin states, affecting gene expression to regulate important processes such as proper development and hormonal signalling pathways.
- We employed transcript profiling, chromatin immunoprecipitation (ChIP), mass spectrometry, yeast two‐hybrid and bimolecular fluorescence complementation protein–protein interaction studies, along with hormone and metabolite profiling and phenotype assessments, to distinguish the SWP73A and SWP73B subunit functions in Arabidopsis.
- We identified a novel subclass of SWI/SNF CRCs defined by the presence of the SWP73A subunit. Therefore, we propose a refined classification of SWI/SNF CRCs in *Arabidopsis*, introducing BRM‐associated SWI/SNF (BAS)‐A (containing SWP73A) and BAS‐B (containing SWP73B) subclasses. The SWP73A‐ and SWP73B‐carrying SWI/SNF CRCs exhibit differential properties, demonstrated by distinct chromatin binding patterns and divergent effects on hormone biosynthesis and metabolism. We additionally found that SWP73A plays a specific role in the regulation of auxin signalling, root development, metabolism and germination that cannot be fully compensated by SWP73B. We recognised that some atypical subclasses of SWI/SNF CRCs may be likely formed in mutant lines with inactivated SWP73 subunits.
- Our study reveals that the duplication of the SWP73 subunit genes contributes to unique and shared functions of SWI/SNF CRC subclasses in the regulation of various processes in *Arabidopsis*.

## Introduction

Switch‐defective/sucrose nonfermentable (SWI/SNF) ATP‐dependent chromatin remodelling complexes (CRCs) are evolutionarily conserved from yeast to plants and mammals. These multisubunit complexes alter histone−DNA interactions and play crucial roles in regulating chromatin structure and transcription. Although the yeast prototype of the SWI/SNF complex was described three decades ago (Stern, 1984), the SWI/SNF structure (Han *et al*., 2020), composition (Mashtalir *et al*., 2020; Yu *et al*., 2020) and functions (Wang *et al*., 2018; Pan *et al*., 2019) are still the subject of thorough study. Three subclasses of SWI/SNF CRCs called cBAF (canonical), pBAF (polybromo) and ncBAF (non‐canonical) exist in humans, while SYD‐associated SWI/SNF (SAS), MINUSCULE‐associated SWI/SNF (MAS) and BRM‐associated SWI/SNF (BAS) have been described in Arabidopsis (J. Guo *et al*., 2022; Fu *et al*., 2023). The subclasses differ in their subunit composition and functional characteristics. In addition to central ATPase and SWI3‐type subunits, one copy of the SWP73/BAF60 (Mashtalir *et al*., 2020; Yu *et al*., 2020) and auxiliary subunits are present in each subclass of SWI/SNF CRCs. Multiplication of *SWP73* and other genes encoding SWI/SNF subunits in humans and plants expands the range of known regulatory pathways targeted by SWI/SNF CRCs (Sarnowska *et al*., 2016; Wang *et al*., 2018; Hernández‐García *et al*., 2022). The Arabidopsis genome encodes two ubiquitously expressed SWP73‐type genes: *SWP73A* (AT3G01890, CHC2/BAF60) and *SWP73B* (AT5G14170, CHC1/BAF60). The *swp73a* mutation provokes early flowering in short‐day conditions, while the *swp73b* mutation causes severe developmental alterations. Mutation of one or both *SWP73A* alleles in the *swp73b* background indicated the existence of unequal functional redundancy between *SWP73A* and *SWP73B*, as evidenced by short lifespan, enhanced dwarfism, altered leaf shape, ectopically positioned meristems of *swp73a*/*SWP73A*; *swp73b/swp73b* sesquimutant plants, and synthetic lethality of *swp73a swp73b* double mutants during embryo development (Sacharowski *et al*., 2015). SWP73A acts as an H3K9me2 reader, triggering an immune response and is involved in the brassinosteroid response, while SWP73B occupancy correlates with the H3K9Ac mark (Jégu *et al*., 2017; Huang *et al*., 2021; Zhu *et al*., 2024). Purification of BRM‐containing BAS SWI/SNF complexes indicated the presence of SWP73A or SWP73B, whereas SWP73B was specifically found in SAS and MAS subclasses carrying SYD and MINU ATPases, respectively (J. Guo *et al*., 2022). Transcriptome analysis of the *swp73b* mutant line indicated altered expression of genes involved in numerous regulatory pathways, including hormone signalling (Bezhani *et al*., 2007; Sacharowski *et al*., 2015). SWP73B regulates chromatin loops (Jégu *et al*., 2014) and is involved in the control of stomatal development (Liu *et al*., 2024). Our previous data suggest that both SWP73 proteins are involved in the regulation of primary and secondary metabolite biosyntheses (Sacharowski *et al*., 2015).

Here, we show that *swp73a* and *swp73b* mutations differentially affect the Arabidopsis transcriptome. Consistently, SWP73A and SWP73B proteins exhibit differential genome‐wide distribution and occupy distinct regions on their target loci, collectively supporting the observed unequal functional redundancy of *SWP73A* and *SWP73B*. Our biochemical study, together with genome‐wide occupancy profiles, indicated that distinct subclasses of SWI/SNF CRCs are defined by SWP73A and SWP73B. Therefore, we propose to update the current SWI/SNF classification by the introduction of BAS‐A (containing SWP73A) and BAS‐B (carrying SWP73B) subclasses. We demonstrated that the SWP73A‐containing BAS‐A and SWP73B‐carrying SWI/SNF CRCs differentially affect hormone biosynthesis and response. Furthermore, the BAS‐A subclass negatively modulates germination dynamics. It also affects root development upon auxin treatment. SWP73A and SWP73B play different roles in the maintenance of metabolic homeostasis. We deduced that in Arabidopsis mutant lines with inactivated subunits of SWI/SNF CRCs, atypical SWI/SNF complexes may be formed and the chromatin localisation of other SWI/SNF subclasses may alter. This study thus considerably broadens the repertoire of molecular functions of SWP73A and SWP73B proteins in Arabidopsis.

## Materials and Methods

### Plant materials and growth conditions

The *Arabidopsis thaliana* L. ecotype Columbia (Col‐0) was utilised as the wild‐type (WT) reference. Three‐week *A. thaliana* seedlings were grown on soil or a ½‐strength Murashige & Skoog medium (½MS) ±0.5% sucrose plates in long‐day, 16 h : 8 h, 22°C : 18°C, light : dark photoperiod or short‐day, 8 h : 16 h, 22°C : 18°C, light : dark photoperiod. Columbia served as a WT background. We used *swp73a‐1*, *swp73b‐1*, *swp73a*/*SWP73A*; *swp73b*/*SWP73B* (to obtain *swp73a*/*SWP73A*; *swp73b*/*swp73b* sesquimutant plants) (Sacharowski *et al*., 2015), *swi3d‐1* (Sarnowski *et al*., 2005) and SALKseq_050120.4 line called *bsh‐3*.

### Cloning and transgenic lines

Coding sequences of SWP73A, SWI3D and BSH were introduced into pDONR207 and pEARLY101 using Gateway and checked by DNA sequencing. The vectors were introduced into *Agrobacterium tumefaciens* strain GV3101 (pMP90) (Koncz & Schell, 1986). Plants were transformed using the floral dip method (Clough & Bent, 1998). Six independent complemented lines for each genotype were selected.

The pSWP73A::SWP73A‐GFP construct was obtained using ‘turbo’ recombineering methods (Hu *et al*., 2019) and introduced into plants using the floral dip method. Primers are listed in Supporting Information Table S1.

### Confocal laser scanning microscopy

Confocal laser scanning microscopy was used on an inverted Nikon C1 confocal system built on TE2000E and equipped with a ×60 Plan‐Apochromat oil immersion objective (Nikon Instruments BV Europe, Amstelveen, the Netherlands). Excitation and detection windows were set as follows: Green Fluorescent Protein (GFP) excitation was achieved using the Sapphire 488 nm laser (Coherent, Santa Clara, CA, USA), and emissions were observed through the 515/530‐nm emission filter.

### PCR genotyping, RNA extraction and RT‐qPCR

Genomic DNA was isolated from leaves using Edward's buffer (Edwards *et al*., 1991) and isopropanol and used for PCR analysis with Taq DNA Polymerase (Takara Bio Europe S.A.S., St Germain en Laye, France) and primers specific for DNA insertions and the target genes (Table S1).

For reverse transcription quantitative polymerase chain reaction (RT‐qPCR), total RNA was extracted from 100 mg of frozen plant tissue using the TRIzol (Sigma) method; the RNA samples were treated with Turbo DNase (Ambion, Austin, TX, USA), and 2.5 μg of the samples was subsequently turned into cDNA with the Transcriptor First Strand cDNA Synthesis Kit (Roche Life Science) using the oligo dT primer. RT‐qPCR was performed with the SYBR Green qPCR Master Mix (Bio‐Rad) and run on a CFX384 Touch instrument (Bio‐Rad). Data were processed in the Cfx manager and exported to Excel. Relative expression was calculated and normalised to the reference gene (UBQ). All primers used in this study can be found in Table S1.

### Chromatin immunoprecipitation

For chromatin immunoprecipitation (ChIP), 2 g of 21 d‐old plants or 0.3 g of dry seeds and 0.5 g of seeds imbibed for 24 h/48 h and different antibodies were used as described previously (Sacharowski *et al*., 2015). GFP‐Trap agarose beads (ChromoTek, Planegg, Germany) against GFP or protein A/G magnetic beads (Thermo Scientific, Waltham, MA, USA) for @SWI3B (1 : 1000; Sarnowski *et al*., 2002) were used for immunoprecipitation. DNA was purified using the Clean‐up DNA Kit (Monarch Spin Kits for DNA Cleanup; NEB, Ipswich, MA, USA). All ChIP experiments were quantified by qPCR with appropriate primers and *TA3* and *Actin7* (AT5G09810) as references (Table S1).

### RNA‐seq and ChIP‐seq analysis

RNA‐seq was performed in two independent biological repeats in 3‐wk‐old plants. Libraries were prepared using Illumina TruSeq Stranded Total RNA with the Ribo‐Zero Plant rRNA Removal protocol and were then single‐end sequenced on HiSeq2500 by the Genome Centre at the Max Planck Institute in Cologne. The quality of the data was assessed using Fastqc. The reads for each sample were aligned to the TAIR10 *A. thaliana* genome using Rna‐Star (Galaxy, v.2.6.0b‐1). Read counts were obtained by implementing HTseq, and subsequent differential expression analyses were performed using DESeq (Bioconductor, Boston, MA, USA). Gene lists were analysed using Lago.

### ChIP‐seq reanalysis

The available Fastq files, deposited by Huang *et al*. (2021) and Jégu *et al*. (2017), were uploaded and processed using the public server (https://usegalaxy.org/). Initial quality control was performed, followed by trimming of the sequences to remove low‐quality bases. The trimmed reads were aligned to the reference TAIR10 genome using Bowtie2 (v.2.4.2) with default settings. Subsequently, peak calling was conducted using the Macs2 peak caller (v.2.1.1) to identify significant binding sites with the following parameters: ‘gsize=119,667,750, bw=300, q=0.01’. The identified peaks were annotated to the genome using the Pavis annotation tool (Huang *et al*., 2013), facilitating the analysis of the genomic context of the binding events.

### Protein precipitation using GFP‐Trap

Material from 20 g plants was ground and proteins were extracted in the IP buffer (50 mM Tris–HCl pH 7.6, 150 mM NaCl, 5 mM MgCl_2_, 10% glycerol, 0.25% TritonX‐100, 1 mM DTT, 10 μM PMSF, 10 μM MG132), complete‐mini protease inhibitor ethylenediaminetetraacetic acid‐free (Roche) supplemented with Viscolase (A&A Biotechnology, Gdansk, Poland). After the centrifugation step of 10 min at 20 000 ***g*** at 4°C, inputs were taken, resuspended in 2× Laemmli buffer and denatured at 95°C for 5 min (for western blot). The rest of the supernatants were added to GFP‐Trap magnetic beads (ChromoTek).

Immunoprecipitated proteins resuspended in 4 M urea 50 mM Tris–HCl pH 8.5 were reduced with dithiothreitol, alkylated with chloroacetamide and digested with trypsin (1 : 100) o/n. Samples were desalted using stage tips with C18 Empore disk membranes (3 M) (Rappsilber *et al*., 2003).

### LC‐MS processing

Immunoprecipitated proteins in 4 M urea 50 mM Tris–HCl pH 8.5 were reduced with DTT, alkylated with chloroacetamide, digested with trypsin (1 : 100) o/n and desalted using stage tips (Rappsilber *et al*., 2003). Peptides were re‐dissolved in 2% Acetonitrile (ACN) and 0.1% TFA, and samples were analysed using an EASY‐nLC 1200 system coupled to a Q Exactive Plus mass spectrometer or using an EASY‐nLC 1000 coupled to a Q Exactive mass spectrometer (all from Thermo Fisher). Peptides (0.5 μg) were separated on 16‐cm fritless silica emitters (75 μm ID; New Objective, Littleton, MA, USA), packed with ReproSil‐Pur C18 AQ 1.9 μm resin (Dr Maisch, Ammerbuch, Germany). Peptides were eluted for 115 min using a segmented linear gradient of 5–95% Solvent B (Solvent A: 0% ACN and 0.1% Formic Acid (FA); Solvent B 80% ACN and 0.1% FA) at 300 nl min^−1^. Mass spectra were acquired in data‐dependent acquisition mode with a TOP15 method. MS spectra were acquired in the Orbitrap analyzer with a mass range of 300–1750 *m/z* at a resolution of 70 000 FWHM and a target value of 3 × 106 ions. Precursors were selected with an isolation window of 1.3 *m/z* (QE Plus) or 2.0 *m/z* (QE). HCD fragmentation was performed at NCE 25. MS/MS spectra were acquired with a target value of 105 ions at a resolution of 17 500 FWHM, a maximum injection time (max.) of 55 ms and a fixed first mass of *m/z* 100. Peptides with a charge of +1, > 6, or with unassigned charge states were excluded from fragmentation for MS2; dynamic exclusion for 30 s prevented repeated selection of precursors.

Data analysis was performed using MaxQuant (v.1.6.3.4) with label‐free quantification (LFQ) and iBAQ enabled, and MS/MS spectra were searched against an *A. thaliana* database (TAIR10_pep_20101214). Perseus (v.1.5.8.5) was used for a statistical analysis of MaxLFQ values, including *t*‐tests and volcano plots (false discovery rate (FDR) < 0.05).

### Germination tests

Four‐month‐old seeds were sown on blue blotter paper, stored in the dark at 4°C for 3 d (stratification) and moved to a chamber with a 16 h : 8 h, light : dark photoperiod at 22°C. Each germination experiment was conducted with at least six replicates (200 seeds from one individual plant grown/Petri dish/genotype).

### Metabolite and hormone measurements

For the analysis of the endogenous hormone level, the aerial parts of 3‐wk‐old WT, *swp73a* and *swp73b* plants were collected, flash‐frozen in liquid nitrogen and subjected to further analysis as described in Sarnowska *et al*. (2023).

Metabolite contents were either determined in the case of primary metabolites in derivatised methanol extracts by gas chromatography mass spectrometry (GC‐MS) using the protocol defined by Lisec *et al*. (2006) or in the case of the glucosinolates by liquid chromatography following the protocol described in Salem *et al*. (2016).

## Results

### The *swp73a* and *swp73b* mutations differentially affect the Arabidopsis transcriptome

**Fig. 1:**
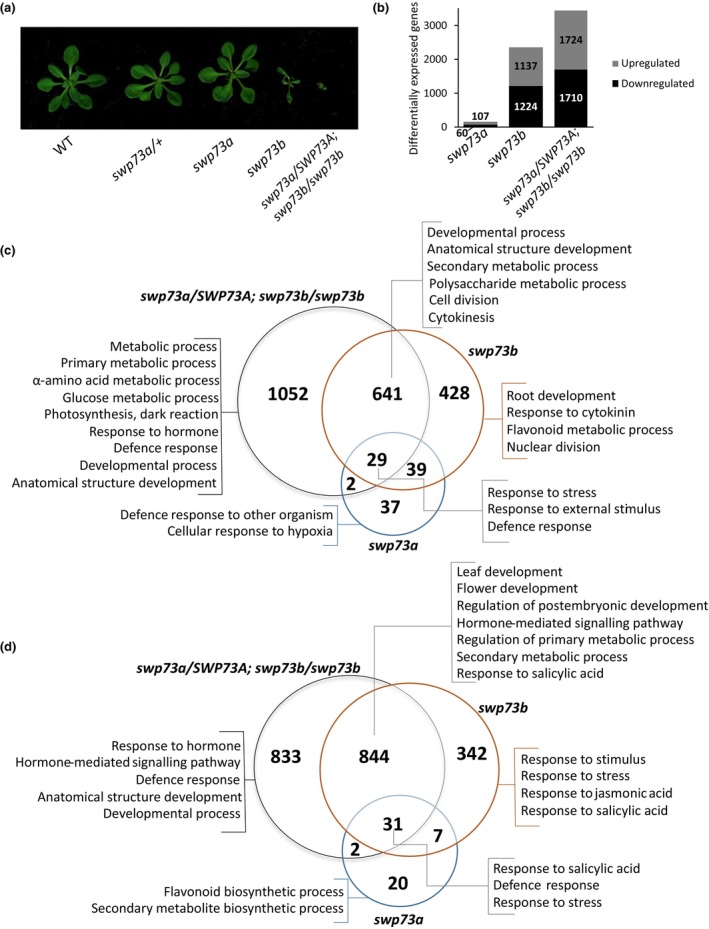
SWP73 subunits of switch‐defective/sucrose nonfermentable (SWI/SNF) chromatin remodelling complexes (CRCs) differentially influence gene expression in Arabidopsis. (a) The phenotypes of 3‐wk‐old wild‐type (WT) plants and T‐DNA insertional lines used in RNA‐Seq analysis. (b) Differentially expressed genes (DESeq2, FC > 1.5, adjusted *P*‐value < 0.05) show increasing numbers among *swp73a*, *swp73b* and *swp73a*/*SWP73A*; *swp73b*/*swp73b*, indicative of unequal functional redundancy of *SWP73A* and *SWP73B*. (c) *swp73a*, *swp73b* mutants, and *swp73a*/*SWP73A*; *swp73b*/*swp73b* sesquimutant exhibit specific and partially overlapping transcriptomic changes among upregulated genes. (d) *swp73a*, *swp73b* mutants, and *swp73a*/*SWP73A*; *swp73b*/*swp73b* sesquimutant exhibit specific and partially overlapping transcriptomic changes among downregulated genes.

We have previously shown that SWP73 subunits of the SWI/SNF CRC modulate several important regulatory processes in Arabidopsis. The *swp73a* mutation leads to early flowering under short‐day conditions; however, it does not affect vegetative growth. By contrast, the Arabidopsis line carrying the *swp73b* mutation exhibits strong developmental alterations, including severe defects in leaves and flowers, male sterility and altered flowering time. The removal of one or two *SWP73A* alleles in the *swp73b* background indicated the existence of unequal functional redundancy of *SWP73A* and *SWP73B*. Namely, the *swp73a*/*SWP73A*; *swp73b/swp73b* sesquimutant plants exhibited a short lifespan, enhanced dwarfism, an altered shape of cotyledons and first true leaves, as well as ectopically positioned meristems, while the *swp73a swp73b* double homozygotes died at the early embryonic stage of development (Fig. 1a; Sacharowski *et al*., 2015).

In order to better understand the molecular basis of the phenotypic alterations observed in the *swp73a/SWP73A*, *swp73a*, *swp73b* and *swp73a*/*SWP73A*; *swp73b*/*swp73b* mutant lines, we conducted transcriptomic analysis. The analysis (DESeq2, absolute log_2_ fold‐change threshold (log_2_FC) ≥ 1.5; FDR‐adjusted *P*‐value < 0.05; Fig. S1a) revealed 30 differentially expressed genes (DEGs) in *swp73a*/*SWP73A* (Table S2), 107 up‐ and 60 downregulated genes in *swp73a* (Fig. 1b; Table S3), 1137 up‐ and 1124 downregulated genes in *swp73b* (Table S4) and 1724 up‐ and 1710 downregulated genes in *swp73a*/*SWP73A*; *swp73b*/*swp73b* plants (Fig. 1b; Table S5). More than 65% of DEGs characteristic of *swp73b* exhibited altered expression in the *swp73a*/*SWP73A*; *swp73b*/*swp73b* line (Fig. 1c,d; Tables S6–S9). Most of them showed the same direction of change – only 0.5% of DEGs (28) in *swp73b* displayed opposite expression patterns in *swp73a*/*SWP73A*; *swp73b*/*swp73b*. One thousand fifty‐two DEGs exhibited specific upregulation in *swp73a*/*SWP73A*; *swp73b*/*swp73b* sesquimutant, while 833 DEGs were specifically downregulated in this line. Among genes specifically upregulated in *swp73a*/*SWP73A*; *swp73b*/*swp73b*, we found ectopically expressed *MIPS1* (Donahue *et al*., 2010), *NF‐YA1*, *NF‐YA5*, *NF‐YA6* (Mu *et al*., 2013) and *SWEET11* (Chen *et al*., 2015) genes involved in seed and embryo developmental processes whose expression terminated during seed dormancy. The Gene Ontology (GO) terms related to both metabolic (including carbohydrate metabolism) and developmental processes were enriched for genes with specifically upregulated expression in the *swp73a*/*SWP73A*; *swp73b*/*swp73b* mutant (Table S8). Among genes specifically downregulated in *swp73a*/*SWP73A*; *swp73b*/*swp73b* sesquimutant, the GO terms related to developmental and defence processes and hormone responses were mainly enriched (Table S9). Genes with elevated expression common for *swp73a*, *swp73b* and *swp73a/SWP73A*; *swp73b*/*swp73b* were associated with stress response and response to another organism, while upregulated genes common for *swp73a/SWP73A*; *swp73b*/*swp73b* and *swp73b* were mainly classified as developmental and primary or secondary metabolism‐related as well as hormone responsive. For genes with downregulated expression common for *swp73a*, *swp73b* and *swp73a*/*SWP73A*; *swp73b*/*swp73b*, the following GO terms were enriched: defence response, response to stress and salicylic acid (SA). The downregulated genes common for *swp73a*/*SWP73A*; *swp73b*/*swp73b* and *swp73b* were classified into developmental, hormone‐ and metabolic‐related GO categories (Fig. 1c,d). Collectively, our study indicated that *swp73a* and *swp73b* mutations have differential impact on the Arabidopsis transcriptome and the observed transcription changes appeared to correlate with the severity of phenotypic traits caused by the particular *swp73* mutations and their combinations including the dosage‐dependent effect of the loss of SWP73A in the background of the *swp73b* mutation.

### SWP73A and SWP73B exhibit differential genome‐wide distribution and occupy distinct regions in their target genes

**Fig. 2:**
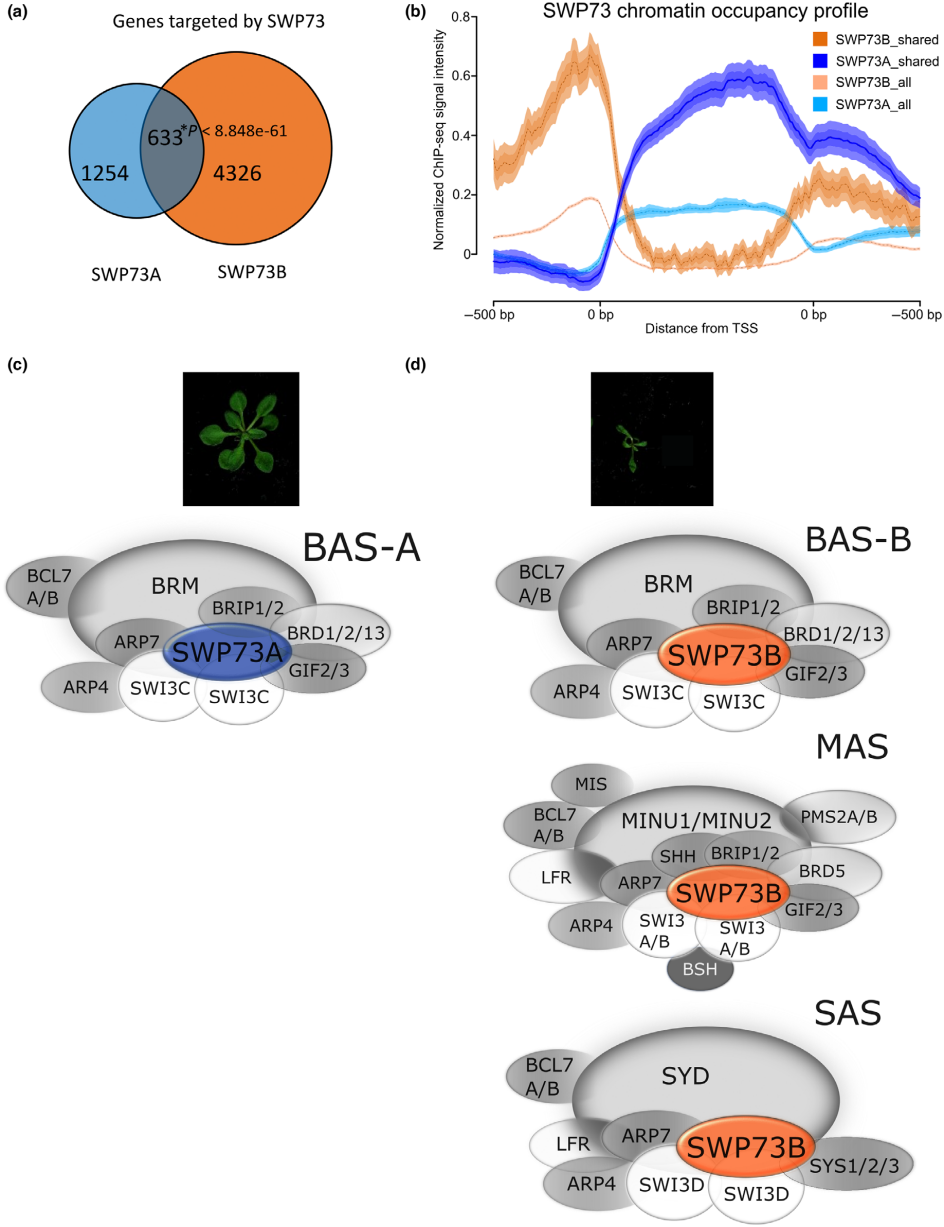
Arabidopsis SWP73A and SWP73B exhibit differential genome‐wide distribution and are present in various subclasses of the switch‐defective/sucrose nonfermentable (SWI/SNF) chromatin remodelling complexes (CRCs) but may cooperate on some target genes. (a) SWP73A and SWP73B bind a set of unique and common targets (reanalysis based on Jégu *et al*., 2017; Huang *et al*., 2021). Asterisks denote statistical significance *P* < 8.848e‐61 determined by the hypergeometric test. (b) Complementary chromatin occupancy profile of SWP73A and SWP73B generated based on all protein‐coding genes from Araport11 and targets shared for SWP73 indicate differential genome‐wide distribution of SWP73 subunits. (c) Graphical representation of the SWP73A‐specific SWI/SNF subclass: BRM‐associated SWI/SNF (BAS) lacking SWP73A impacts plant growth under standard, long‐day conditions. (d) Graphical representation of the SWP73B‐specific SWI/SNF subclasses: BAS, MINUSCULE‐associated SWI/SNF (MAS), and SYD‐associated SWI/SNF (SAS) lacking SWP73B impacts plant growth under standard, long‐day conditions.

We reanalysed the existing datasets for global occupancy profiles of SWP73A and SWP73B (Jégu *et al*., 2017; Huang *et al*., 2021). SWP73A was found to target 1887 genes while SWP73B targeted 4959 loci and thus appeared to be more widespread in the Arabidopsis genome (Fig. 2a; Tables S10–S13). Six hundred thirty‐three loci were commonly bound by SWP73A and SWP73B, representing *c*. 33% of all SWP73A and 12% of SWP73B targets. The commonly bound genes are mainly classified into general processes, including stress response, response to hormone‐ and metabolism‐related GO terms (Tables S14–S19). The comparison between RNA‐seq data and the SWP73A occupancy resulted in the identification of 15 genes with upregulated and four genes with downregulated expression in *swp73a*, which were directly regulated by SWP73A. An analogous analysis conducted for SWP73B indicated that 284 genes upregulated in *swp73b* are directly regulated by SWP73B while 379 downregulated in *swp73b* are targeted by SWP73B (Fig. S1b). These DEGs were classified into general GO terms, including response to stimulus, hormone and metabolic processes among both up‐ and downregulated genes in *swp73b*, which appeared to be directly targeted by SWP73B (Tables S16, S17). Given the relatively low common set of genes, we next compared the binding of SWP73A and SWP73B with DEGs in the *swp73a*/*SWP73A*; *swp73b*/*swp73b* line. We found 165 up‐ and 161 downregulated genes in *swp73a*/*SWP73A*; *swp73b*/*swp73b* plants that are directly controlled by SWP73A, while 449 up‐ and 466‐downregulated genes were direct targets of SWP73B, mainly being classified under hormone‐ and metabolite‐related GO classes. However, the number of GO terms for SWP73A was about half of those enriched for SWP73B. This finding suggests that SWP73 subunits may play both specific and overlapping roles in regulating their target genes, as indicated by our transcriptomic analysis, and that the function of SWP73A is more highlighted in the absence of SWP73B (Fig. S1b; Tables S16–S19). To test this hypothesis further, we inspected the chromatin regions being occupied by both proteins. We found that SWP73A and SWP73B have distinct genome‐wide chromatin occupancy profiles across the promoter regions and the gene body (called by us ‘all’). SWP73B enrichment was mainly observed in the promoter and transcription start site (TSS) regions, while SWP73A targeted gene bodies even on genes bound simultaneously by both SWP73 proteins, called ‘shared’ (Fig. 2b).

### Distinct subclasses of the SWI/SNF chromatin remodelling complexes are defined by SWP73A and SWP73B

Given the reported existence of various SWI/SNF subclasses in Arabidopsis (J. Guo *et al*., 2022; Fu *et al*., 2023; Stachula *et al*., 2023), we next investigated the composition of the SWI/SNF subclasses containing a particular SWP73 subunit using transgenic plants expressing SWP73A‐ or SWP73B‐YFPHA complementing the *swp73a* or *swp73b* mutations, respectively (Fig. S2). We performed five independent liquid chromatography tandem mass spectrometry (LC‐MS/MS) measurements (Tables S20–S23) and found that SWI3C and BRM were significantly enriched in SWP73A samples (Fig. 2c; Table S20). By contrast, SWP73B co‐purified with all four ATPases (BRM, SYD, MINU1 and MINU2) and all four SWI3 subunits (SWI3A, B, C, D; Fig. 2d; Table S21). The DNA‐binding bromodomain‐containing proteins, BRD1, BRD2 and BRD13 (Yu *et al*., 2021; Stachula *et al*., 2023) and BRAHMA‐interacting protein 2, a direct interactor of SWP73‐type subunits (Yu *et al*., 2020), co‐purified with both SWP73A and SWP73B proteins. The following subunits were observed with both SWP73: ARP4, ARP7, and BCL7B, whereas BCL7A interacted with SWP73B only, consistent with previously published data (Vercruyssen *et al*., 2014; Yu *et al*., 2021; J. Guo *et al*., 2022; Fu *et al*., 2023; Stachula *et al*., 2023). The specific role of SWP73A in the BRM‐containing SWI/SNF complex class (BAS), the only class of SWI/SNF complexes where SWP73A was present, and the general role of SWP73B, were confirmed by SWI3D and BSH LC‐MS/MS analysis. We confirmed that SWI3D is a subunit of the SYD‐specific SWI/SNF complex (SAS), carrying SWP73B and lacking SWP73A (Table S22). Except for SYD and SWP73B, the SWI3D‐containing complexes carried ARP4, ARP7, SYS1‐3, BCL7A and BCL7B, GIF2 and LFR subunits. The LC‐MS/MS data obtained using BSH as a bait clearly indicated that this subunit is associated with the MINU‐SWI/SNF classes (MAS), where it occurs together with SWP73B (Table S23). Proteomics analyses demonstrated that SWP73B plays an independent role in the assembly of SYD‐specific (SAS) and MINU‐specific (MAS) SWI/SNF classes, both SWP73‐defined subclasses of BRM‐SWI/SNF complexes, which we called BAS‐A (containing SWP73A) and BAS‐B (carrying SWP73B).

To test whether the findings from proteomic analysis reflect chromatin occupancy patterns and functional diversification, we compared chromatin occupancy meta‐profiles for SWP73A, SWP73B, BRM and SYD. We used three sets of genes: those co‐occupied by both SWP73 proteins (shared), and those uniquely occupied by either SWP73A (SWP73A‐specific) or SWP73B (SWP73B‐specific). Reanalysis of ChIP‐Seq data (Li *et al*., 2016) revealed that the maximum of SYD binding appears slightly upstream of the TSS with a rapid decline in the gene body, whereas the maximum of BRM binding is located within the gene body shortly after the TSS, showing a milder descent and re‐enrichment at the 3'UTR (Fig. S3a). The occupancy profile of SYD very closely matched that of SWP73B, with significant enrichment in the promoter and near the TSS region. Interestingly, BRM partially overlapped with both SWP73 proteins: in the promoter region with SWP73B, and in the gene body and 3'UTR regions with SWP73A. This pattern was confirmed on genes uniquely bound by SWP73A (SWP73A‐specific), where its occupancy, along with BRM, increased in the TSS and gene body regions compared with the global profile (Fig. S3b). By contrast, SWP73A showed no association with genes specifically occupied by SWP73B (SWP73B‐specific), whereas both BRM and SYD exhibit significant enrichment (Fig. S3c). The specificity of the BAS‐A and BAS‐B subclasses of the SWI/SNF complex is further supported by the analysis of peaks specific to BRM, SYD, SWP73A and SWP73B annotated across the genome (Fig. S4a; Table S24). We found that, in the promoter region, the peaks of BRM and SYD overlap almost exclusively with SWP73B, indicating that BAS‐B and SAS complexes are involved in the regulation of this region. Conversely, within the gene body, BAS‐A, along with BAS‐B and SAS complexes, may co‐regulate gene expression (Fig. S4a,b). Interestingly, a detailed analysis of the occupancy profiles of SWP73A, SWP73B and BRM further supported the existence of distinct BAS‐A and BAS‐B subclasses. As we showed, SWP73A and SWP73B exhibit mutually exclusive binding patterns, yet both overlap with BRM (Fig. S4b) and 16 out of 19 genes with altered expression in the *swp73a* line are targeted directly by BRM. Overall, these findings along with our biochemical study highlight the functional diversification of SWI/SNF complexes based on the particular SWP73 subunit.

One puzzling question was the co‐purification of SWP73A with SWP73B and vice versa. Based on the LFQ value difference between SWP73A and SWP73B, the probability that SWP73A and SWP73B occurred together in the same complex is *c*. 1 in 250. Considering the complementary occupancy profile of SWP73, we rather anticipate that SWP73A and SWP73B may interact in a temporary manner. However, we confirmed the possibility of the existence of such interactions using both bimolecular fluorescence complementation (BiFC) and yeast two‐hybrid (Y2H) assays (Fig. S5a,b), consistent with recent findings that SWP73B co‐precipitated with SWP73A (J. Guo *et al*., 2022).

### SWP73A‐containing BAS‐A and SWP73B‐carrying SWI/SNF CRCs differentially affect hormonal response and hormone biosynthesis in Arabidopsis

The detailed examination of transcriptomics and genome‐wide occupancy data indicated that both SWP73A and SWP73B are involved in hormonal regulation (Tables S2–S18, S25). SWP73A directly binds to genes involved in the biosynthesis and signalling of multiple hormones, including jasmonic acid, gibberellic acid (GA), SA, abscisic acid, brassinosteroids, ethylene and auxin (Fig. S6a; Table S26). Similarly, SWP73B directly regulates genes associated with abscisic acid, auxin, cytokinin, SA and the biosynthesis of ethylene, jasmonic acid and gibberellin (Fig. S6b; Table S27). These findings suggest that both SWP73s may play a significant role in hormonal response and hormone biosynthesis in Arabidopsis.

**Fig. 3:**
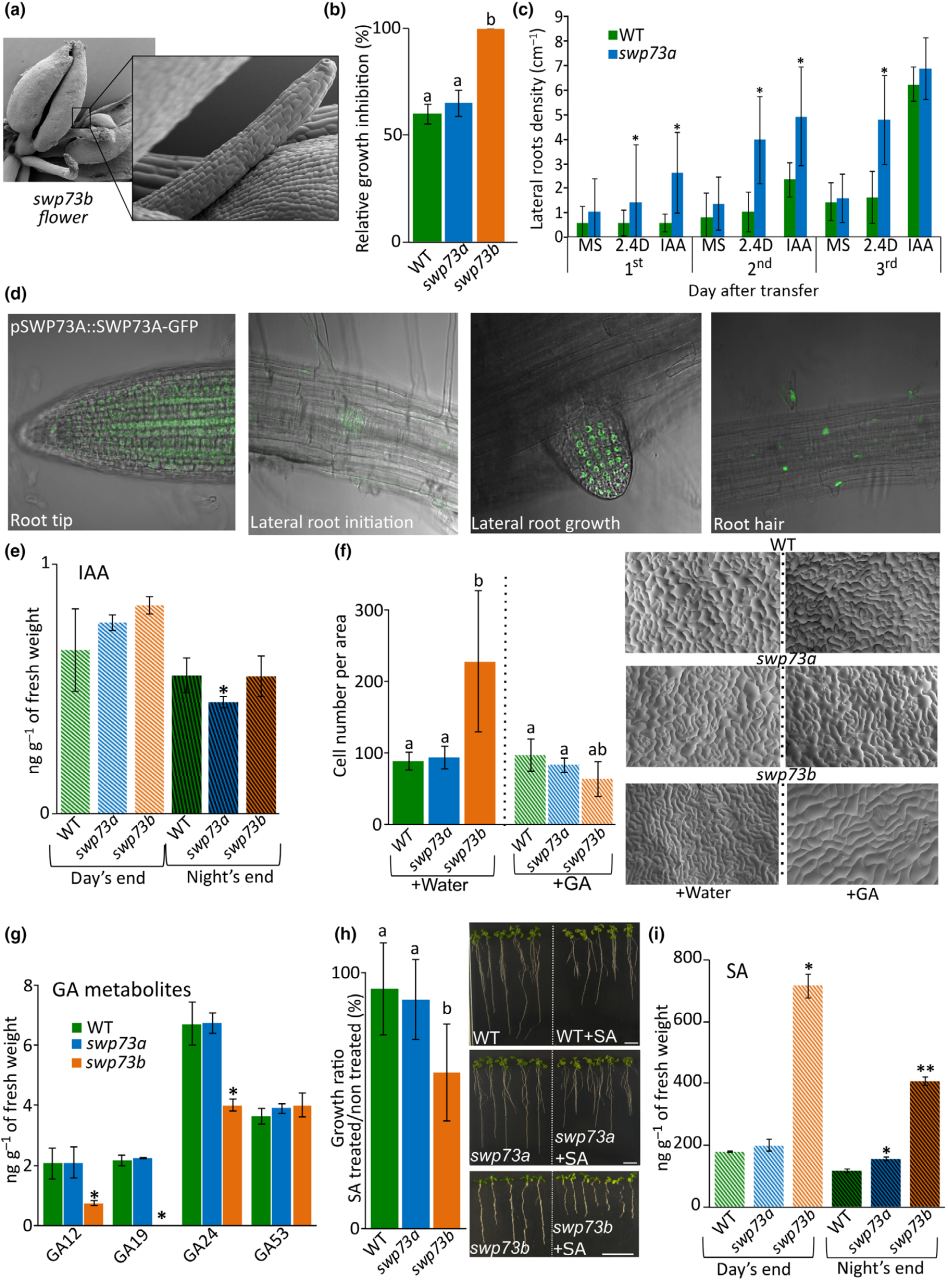
*SWP73A* and *SWP73B* differentially impact the Arabidopsis response to selected phytohormones and hormone biosynthesis. (a) Pinoid‐like structures occurring in *swp73b* flowers. (b) *swp73b* plants exhibit hypersensitivity to 2,4‐D demonstrated as enhanced growth inhibition. Letters correspond to statistical significance determined by the Kruskal–Wallis ANOVA with *post hoc* Dunn's test (*P* < 0.001). (c) *swp73a* mutation causes increased frequency of lateral root formation in response to indole‐3‐acetic acid (IAA) and 2,4‐D. Charts represent lateral root numbers in the wild‐type (WT) and *swp73a* counted from first to third day after transferring 6‐d‐old seedlings from a ½‐strength Murashige & Skoog medium (½MS), to ½MS, ½MS with 50 μM IAA or 1 mM 2,4‐D. Error bars denote the SD, while asterisks indicate statistical significance (*P*‐value < 0.05, Student’s *t*‐test) (d) SWP73A protein is present in the root meristem zone, lateral root (including root primordia) and root hair. (e) Indole‐3‐acetic acid level in the WT, *swp73a* and *swp73b* mutants. Error bars denote the SD, while asterisks denote statistically significant enrichment compared with WT plants (*t‐*test, the asterisk denotes *P*‐value < 0.05). (f) *swp73b* plants exhibit specific cell cycle alterations demonstrated by the increased cell number in leaves epidermis that are reversed by the gibberellin spraying (100 mM GA_(4 + 7)_). Counted from at least 10 different leaves for each sample. Letters correspond to statistical significance determined by the Kruskal–Wallis ANOVA with Dunn's *post hoc* test. On the right, representative images of leaf surface collected from 3‐wk‐old WT, *swp73a*, *swp73b* treated or nontreated with gibberellic acid (GA) through the life cycle are given. (g) *swp73b* affects gibberellin metabolite levels. Error bars denote the SD, while asterisks denote statistically significant enrichment compared with WT plants (*t*‐test, the asterisk denotes *P*‐value < 0.05). (h) *swp73b* is hypersensitive to SA treatment. Growth ratio of *swp73a* and *swp73b* in response to 5 μM salicyclic acid (SA). Error bars denote the SD, while letters correspond to statistical significance determined by the Kruskal–Wallis ANOVA with *post hoc* Dunn’s test (*P* < 0.05). (i) SA amount in the WT, *swp73a* and *swp73b* mutants. Error bars denote the SD, while asterisks denote statistically significant enrichment compared with WT plants (*t*‐test; *, *P*‐value < 0.00005; **, *P*‐value < 0.000005).

These data, together with phenotype alterations observed in the *swp73b* mutant, such as the presence of pinoid‐like structure in *swp73b* flowers (Fig. 3a; Sacharowski *et al*., 2015), prompted us to examine the response of the *swp73a* and *swp73b* mutants to 2,4‐dichlorophenoxyacetic acid (2,4‐D) and to indole‐3‐acetic acid (IAA).

*swp73a* responded to 2,4‐D similarly to WT in terms of main root growth inhibition, while the *swp73b* mutant displayed a total inhibition of root and stem growth and forming instead pin‐like structures that demonstrate auxin hypersensitivity (Figs 3b, S7a). Furthermore, the main root of *swp73b* is nearly four times shorter than that of WT and *swp73a* (Fig. S7b). By contrast, a detailed analysis of *swp73a* plants cultivated on medium supplemented with IAA/2,4‐D revealed that they form more lateral roots than WT (Fig. 3c), suggesting increased lateral root primordia in *swp73a*; however, this requires additional careful study. *swp73a* exhibited similar lateral root density to WT under control growth conditions. The primary root length of *swp73a* remains unchanged upon auxin treatment (Fig. S7c). This is consistent with SWP73A targeting a class of genes involved in root development (Table S26). Therefore, we used the pSWP73A::SWP73A‐GFP *swp73a* line and found that the SWP73A protein is present in the root tip, nascent root primordium cells, tips of lateral roots and root hairs (Fig. 3d). The SWP73A localisation changed upon auxin treatment (Fig. S7d) collectively supporting an important role of SWP73A in lateral root formation in an auxin‐responsive manner.

These findings regarding the role of SWP73A in lateral root formation and its localisation in response to auxin treatment are further contextualised by the metabolic pathways involved in auxin synthesis, particularly the IAA pathways. IAA is mainly synthesised via the indole‐3‐pyruvic acid (IPyA) and indole‐3‐acetamide (IAM) pathways, with the IPyA pathway being dominant in *A. thaliana* (Mashiguchi *et al.*, 2011; Cao *et al.* 2019; Tillmann *et al*., 2022; Fig. S7e). The measurement of IAA and its intermediates (Figs 3e, S7f–j) indicated that the IAA level was slightly decreased only in *swp73a* at the end of the night (Fig. 3e), consistent with a decreased level of IAM in the *swp73a* mutant (Fig. S7g). The precursor of IAA synthesis – tryptophan – was unchanged in both *swp73* mutants (Fig. S7f). Simultaneously, *swp73b* showed elevated levels of IAA conjugated with amino acids (Fig. S7h,i) and the major IAA catabolite – OxIAA (Pěnčík *et al*., 2013; Fig. S7j), despite no changes in bioactive auxin levels, suggesting that *swp73b* may have enhanced auxin catabolism; however, it requires additional precise study.

Auxin regulates its own biosynthesis and transport through feedback mechanisms (Yu *et al*., 2022). Given the *swp73a* phenotypic alterations upon auxin treatment and decreased level of bioactive IAA in this mutant, we checked the presence of SWP73A on genes involved in auxin transport (PINs) and conjugation (*GH3.17*, involved in the control of root meristem size (R. Guo *et al*., 2022) and *YDK1/GH3.2*). We found that SWP73A directly targets *PIN1*, *PIN4*, *PIN7*, *GH3.17* and *YDK1* genes (Fig. S8a) and that the *swp73a* mutant plants exhibited downregulated expression of *PIN1* and *PIN4* while *PIN7*, *GH3.17* and *YDK1* genes were upregulated 4 h after auxin treatment (Fig. S8b), providing a functional link with the observed SWP73A localisation pattern in roots. This finding is consistent with previous data showing that BRM is involved in root development through the direct regulation of *PINs* expression (Yang *et al*., 2015). Thus, a close inspection of the occupancy of these loci by BRM indicated that both subunits of the BAS‐A subclass of SWI/SNF CRCs may co‐regulate the expression of these genes (Fig. S8b). Overall, our analysis suggests that SWP73A plays an important role in the control of auxin homeostasis and in root development upon auxin treatment, which cannot be compensated by SWP73B‐containing SWI/SNF CRCs in the *swp73a* mutant.

Given the established crosstalk between auxin and cytokinin in regulating root and shoot development (Moubayidin *et al*., 2009), we next examined the phenotype of *swp73a* and *swp73b* in response to 6‐Benzylaminopurine (BAP). *swp73b* plants were more sensitive to 1 μM BAP and failed to accumulate anthocyanins, unlike WT and *swp73a* (Fig. S9a,b). Phenotypic analysis indicates the overaccumulation of cytokinins in *swp73b*. Indeed, the measurement of cytokinins showed an increased accumulation of isopentyladenine and its metabolites (isopentenyladenine riboside and N‐glucosides isopentenyladenine‐9‐glucoside) in the aerial part of *swp73b* (Fig. S9c). This, together with the findings of Jégu *et al*. (2015), supports the involvement of SWP73B‐containing classes of SWI/SNF complexes in cytokinin biosynthesis.

We next investigated the response of *swp73* mutants to exogenous gibberellin. The s*wp73a* line responded like WT plants, while the bushy phenotype of *swp73b* was partially reversed (Fig. S10), although *swp73b* plants were still unable to flower under short‐day conditions. Phenotype analysis using a scanning electron microscope indicated that the *swp73b* mutation resulted in more frequent cell divisions, which were reversed upon GA treatment (Fig. 3f). The enhanced cell division phenomenon was in keeping with the cell cycle progression markers observed in transcript profiling, with cytokinesis and DNA recombination GO terms being enriched among the *swp73b* upregulated genes (Table S6). Moreover, consistent with the dwarf phenotype of the *swp73b*, we found significantly lower amounts of GA12 and GA24 and a complete absence of GA19 in this line (Fig. 3g) supporting an important SWP73B function in GA biosynthesis and signalling. No changes in GA53 levels were detected.

The *swp73b* was more sensitive in terms of root length to 5 μM SA than the WT and *swp73a* (Fig. 3h). The *swp73b* exhibited about seven‐fold SA accumulation at the end of the day and about four‐fold at the night's end, whereas *swp73a* plants demonstrated only mild SA overaccumulation at the end of the night (Fig. 3i). Together with the hypersensitivity of *swp73b* plants to SA treatment, this result suggested a more pronounced role of SWP73B‐containing SWI/SNF complexes in SA biosynthesis and signalling.

**Fig. 4:**
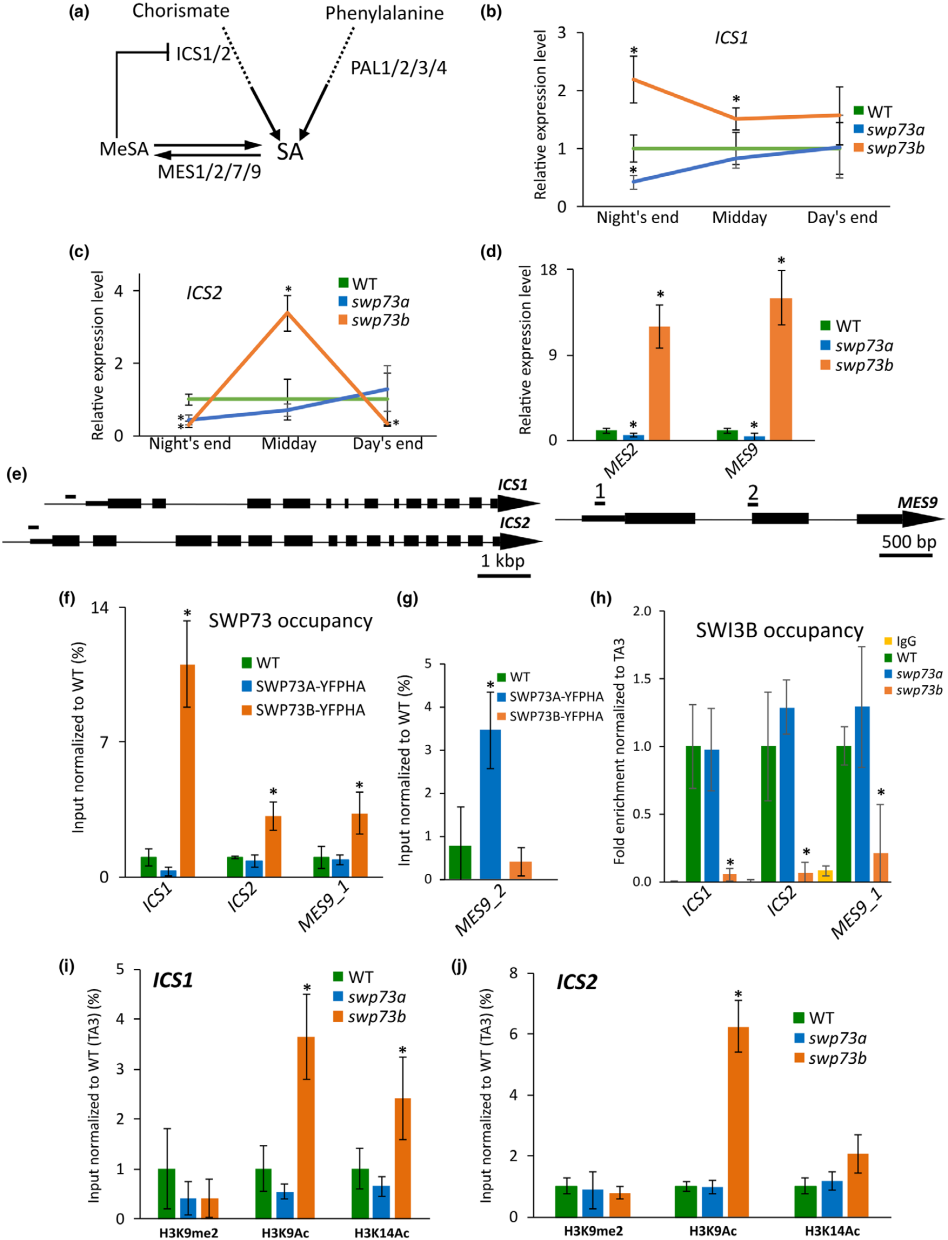
SWP73A and SWP73B play differential roles in salicylic acid (SA) biosynthesis in Arabidopsis. (a) Simplified scheme of SA biosynthesis pathway in Arabidopsis. Solid arrows indicate a single‐step enzymatic reaction leading to the formation of the subsequent metabolite. Dashed lines represent a multistep process not detailed in the scheme. Blunt‐ended arrows denote an inhibitory process. (b) Differential effect of *swp73a* and *swp73b* on the expression of *ICS1*, (c) *ICS2* and (d) *MES2* and *MES9* genes. Data are shown as the mean ± SD (*n* = 3). Error bars denote the SD, while the asterisks show statistical significance *P*‐values by the student's *t*‐test (*P* < 0.05). (e) Scheme of the genes involved in SA biosynthesis with amplicon positions corresponding to (f–j). (f) SWP73B binds directly the *ICS1*, *ICS2* and *MES9* loci. (g) SWP73A directly binds MES9 gene body. Data are shown as the mean ± SD (*n* = 3). The asterisks represent *P*‐values by Student's *t*‐test (*P* < 0.05). Samples were collected at the end of the night. (h) SWI3B occupancy on the regulatory region of genes involved in SA biosynthesis is affected in the *swp73b* mutant. Error bars denote the SD, while asterisks indicate statistical significance (*n* = 3, *P*‐value < 0.05, Student's *t*‐test). *swp73b* mutation causes alterations in histone modification presence on (i) *ICS1* and (j) *ICS2* loci. Error bars denote the SD, while asterisks indicate statistical significance (*n* = 3, *P*‐value < 0.05, Student's *t*‐test). Samples for (f, h–j) were collected at midday. ICS, isochorismate synthase.

There are two pathways responsible for SA biosynthesis. Chorismate, synthesised by isochorismate synthase (ICS), is the main source, leading to the production of *c*. 90% of SA in *Arabidopsis* (Garcion *et al*., 2008; Lefevere *et al*., 2020; Fig. 4a), while a minor route is catalysed by phenylalanine ammonia‐lyase (PAL).

The Arabidopsis genome encodes two homologues of *ICS* (*ICS1* and *ICS2*) and four of *PAL* (*PAL1*, *PAL2*, *PAL3* and *PAL4*). We found a two‐fold downregulation of *ICS1* expression in the *swp73a* mutant at the end of the night, while the *swp73b* line exhibited two‐fold *ICS1* upregulation at the end of the night, which decreased during the day (Fig. 4b). The *ICS2* expression was downregulated in the *swp73a* and *swp73b* lines at the end of the night and strongly upregulated 6 h after dawn in the *swp73b* mutant. However, in the *swp73b* mutant, the *ICS2* expression dropped again at the end of the day (Fig. 4c). The *swp73* mutants additionally exhibited differential changes in expression levels of *MES2* and *MES9* genes encoding methyltransferases converting methyl salicylate (MeSA) to SA (Fig. 4d). *MES2* and *MES9* were about two‐ to three‐fold downregulated in the *swp73a* mutant, whereas they were strongly induced in the *swp73b* mutant (12‐ and 15‐fold, respectively) at the end of the night. Decreased expression of *MES2* and *MES9* in the *swp73a* mutant could possibly lead to increased levels of MeSA and consequently to downregulation of *ICS1* and *ICS2* expression, which is observed in *swp73a* at the end of the night, indicating the involvement of SWP73A‐containing BAS‐A complexes in the feedback loop controlling SA biosynthesis in Arabidopsis (Fig. 4b–d). The role of SWP73B in the proper maintenance of the regulatory feedback loop controlling SA biosynthesis and catabolism appeared more pronounced given the strong upregulation of *MES2* and *MES9* genes and the overaccumulation of SA at the night's end.

We found that SWP73B binds to the promoter region of *ICS1* (−150 bp to TSS), *ICS2* and *MES9* (5′UTR), which resulted in a 3‐ to 10‐fold enrichment of expression (Fig. 4e,f). SWP73A was not present on the promoter regions of these loci, but targeted the *MES9* gene body in plants collected at the night's end (Fig. 4g).

To determine whether the incorporation of other SWI/SNF subunits/subclasses depends on SWP73s, we performed ChIP‐qPCR analysis using anti‐SWI3B antibodies in both *swp73a* and *swp73b* backgrounds and found that the SWI3B binding to *ICS1*, *ICS2* and *MES9* loci is reduced in the absence of the SWP73B protein (Fig. 4h). Given that SWP73A may function as an H3K9me2 modification reader (Huang *et al*., 2021), and BAF60c, a human counterpart of Arabidopsis SWP73, is associated with the H3K9 acetylation (Forcales *et al*., 2012), we next assessed the chromatin status on SA biosynthesis genes in both *swp73* mutants by monitoring H3K9me2, H3K9Ac and H3K14Ac in the region recognised by SWP73B. Our results indicate that the lack of SWP73B leads to increased H3K9Ac and H3K14Ac enrichment on the proximal promoter of *ICS1* and H3K9Ac on the TSS region of *ICS2*, consistent with elevated expression of *ICS1* and *ICS2* in the *swp73b* line (Fig. 4i,j). We showed that SWP73B can bind to active chromatin regions, determined by H3K9Ac, and function as a repressive factor, influencing the transcription of genes involved in SA biosynthesis.

We next evaluated the rapid response to SA and found that SA treatment resulted in two‐fold *ICS2* downregulation in WT plants and dramatic (*c*. 10‐fold) downregulation in *swp73b*, where the expression dropped below the values observed in WT plants. By contrast, the *ICS2* expression remained unchanged in the case of *swp73a*. SA can modulate the expression of *Gibberellic Acid Stimulated in Arabidopsis* (*GASA*) genes, specifically *GASA4* and *GASA5* (Alonso‐Ramírez *et al*., 2009; Zhang *et al*., 2009; Zhang & Wang, 2011), making them relevant candidates for studying SA perception in the context of the *swp73* mutants. *GASA4* expression in untreated *swp73b* plants showed a 1.5‐fold upregulation, which was abolished by SA treatment, returning to levels similar to WT. The *c*. 1.5‐fold upregulation of *GASA5* in *swp73a* and two‐fold in *swp73b* were also abolished by SA treatment (Fig. S11). Collectively, our results indicate differential roles of SWP73A‐containing BAS‐A and SWP73B‐carrying subclasses of SWI/SNF CRCs in SA biosynthesis and perception.

**Fig. 5:**
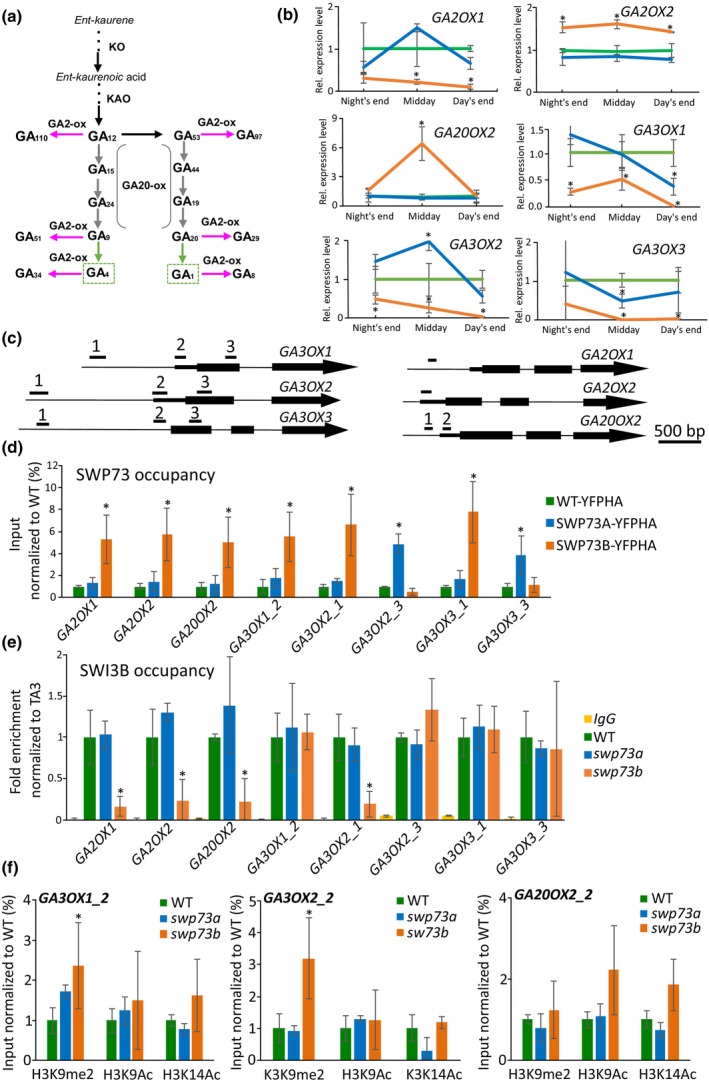
*swp73a* and *swp73b* mutations lead to defects in gibberellin biosynthesis and catabolism. (a) Simplified scheme of gibberellin biosynthesis and catabolism pathway in Arabidopsis. Green colour represents bioactive form of gibberellin, while magenta indicates inactive form. (b) The *swp73a* and *swp73b* mutations differentially affect the expression of genes involved in gibberellin biosynthesis. Error bars denote the SD, while asterisks indicate statistical significance (*n* = 3, *P*‐value < 0.05, Student's *t*‐test). (c) Scheme of the genes involved in GA biosynthesis with amplicon positions corresponding to (d–f). (d) SWP73A and SWP73B bind to the regulatory region of genes involved in gibberellin biosynthesis. Error bars denote the SD, while asterisks indicate statistical significance (*n* = 3, *P*‐value < 0.05, Student's *t*‐test). (e) SWI3B binding *to* loci of genes involved in gibberellin biosynthesis is differentially affected in *swp73a* and *swp73b* plants. Error bars denote the SD, while asterisks indicate statistical significance (*n* = 3, *P*‐value < 0.05, Student's *t*‐test). (f) *swp73a* and *swp73b* lines exhibit differential effects on chromatin status on loci of genes involved in gibberellin biosynthesis. Error bars denote the SD, while asterisks indicate statistical significance (*n* = 3, *P*‐value < 0.05, Student's *t*‐test).

Upregulation of *GASA5* in *swp73a* and *swp73b* suggests alterations in gibberellin‐related pathways in these mutants. Considering genome‐wide and phenotypic data, we decided to examine gibberellin biosynthesis. Expression of two cytochrome P450 monooxygenases: *ENT*‐*KAURENE OXIDASE 1* (*KO1/GA3*) *ent*‐kaurenoic acid oxidase (KAO) and *ENT‐KAURENE SYNTHASE 1* (*KS1/GA2*) catalysing the first steps of GA biosynthesis (Sun, 2008; Helliwell *et al*., 2011; Fig. 5a), was unchanged in the *swp73a* and *swp73b* mutants compared with that in the WT control (Fig. S12a).

However, *swp73b* mutation resulted in a 2.5‐fold downregulation of *GIBBERELLIN 2‐OXIDASE 1* (*GA2OX1*) and *c*. 1.5‐fold upregulation of *GIBBERELLIN 2‐OXIDASE 2* (*GA2OX2*) expression. The expression of *GA20OX2* was elevated about six‐fold in *swp73b* plants only at the middle of the day. We also observed a 2‐ to 10‐fold downregulation of *GIBBERELLIN 3 BETA‐HYDROXYLASE* (*GA3OX1–3*) family genes (Fig. 5b), crucial for the synthesis of biologically active GA1 and GA4. The *swp73a* line demonstrated only mild alterations in the expression levels of *GA3OX1–3* genes, indicating a major function of SWP73B‐containing SWI/SNF complexes in the control of GA biosynthesis.

We also examined the expression levels of genes encoding the GA repressors‐*RGL2* and *RGL3* and GA receptors – *GID1A*, *GID1B* and *GID1C*. We found that *RGL2* is upregulated in *swp73b* and minorly elevated in the *swp73a* mutant, whereas *RGL3* is only significantly downregulated in *swp73b* (Fig. S12b). Among GID1 receptors, only *GID1B* expression was affected in *swp73b*.

We found that SWP73B directly binds all six GA biosynthesis loci, whose expression was affected by the *swp73b* mutation (Fig. 5c,d). We detected SWP73B binding to the proximal promoter of *GA2OX1* (−350 bp to TSS), *GA20OX2* (−100 bp to TSS) and *GA3OX3* (−150 bp to TSS), as well as the 5′UTR/TSS regions of *GA2OX2*, *GA3OX1* and *GA3OX2*. SWP73A targeted *GA3OX2* (+300 after TSS) and *GA3OX3* (+200 after TSS) in their gene bodies, supporting their discretely altered expression levels in the *swp73a* mutation. We showed that SWP73B is necessary for SWI3B binding in the *GA2OX1*, *GA2OX2*, *GA20OX2* and *GA3OX2* promoter regions, whereas SWI3B binding to *GA3OX1* and *GA3OX3* promoters was unaffected by the *swp73b* mutation (Fig. 5e).

The H3K9me2 repressive mark was enriched at the *GA3OX1* and *GA3OX2* loci, consistent with their reduced expression in the *swp73b* mutant (Fig. 5f). By contrast, the activating chromatin marks H3K9Ac and H3K14Ac were increased at the *GA20OX2* promoter in the *swp73b* mutant, correlating with the upregulated expression of the genes. Our data collectively indicate that both SWP73s target GA‐biosynthesis genes, thus comprising a major function of SWP73B.

### The BAS‐A subclass of SWI/SNF chromatin remodelling complexes negatively modulates germination dynamics via regulation of *GA3OX* gene expression

**Fig. 6:**
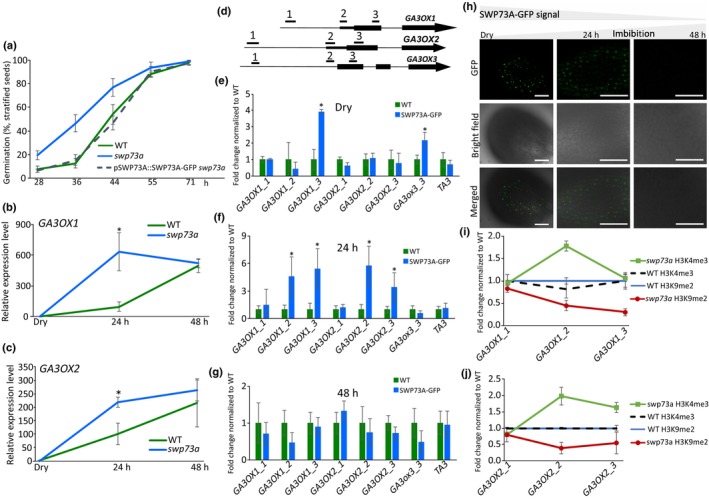
BRM‐associated SWI/SNF (BAS)‐A complex regulates germination speed through the repression of *GA3OX1* and *GA3OX2* expression in Arabidopsis. (a) Seed germination of the wild‐type (WT) and the *swp73a* mutant and the SWP73A::SWP73A‐GFP *swp73a* line, after 2 d of stratification. Germination was scored at given time point. Error bars show the SD of four biological replicates. *, *P* < 0.05. (b) Expression of *GA3OX1* is affected in *swp73a*. (c) Expression of *GA3OX2* is affected in *swp73a*. Error bars denote the SD, while asterisks indicate statistical significance (*n* = 3, *P*‐value < 0.05, Student’s *t*‐test). (d) Scheme of the genes involved in gibberellic acid (GA) biosynthesis with amplicon positions corresponding to (e–g, i, j). SWP73A occupancy measured by chromatin immunoprecipitation (ChIP)‐qPCR alters in dry seeds (e), and imbibed seeds after 24 h (f) and 48 h (g). Values were normalised to WT, and TA3 was used as a control. Error bars denote the SD, while asterisks indicate statistical significance (*n* = 3, *P*‐value < 0.05, Student's *t*‐test). (h) Confocal microscope analysis of SWP73A‐GFP signal indicates the presence of SWP73A protein in dry seeds and imbibed seeds after 24 h, whereas after 48 h SWP73A was not detected. Bar, 50 μm. (i) *swp73a* affects H3K9me2 and H3K4me3 occupancy on *GA3OX1* and (j) *GA3OX2*. In *swp73a*, H3K4me3 was indicated by a green line and rectangles, while H3K9me2 was denoted by a dark red line and circles. Values were normalised to WT and to *TA3.* Error bars denote the SD, while asterisks indicate statistical significance (*n* = 3, *P*‐value < 0.05, Student's *t*‐test).

Considering the altered *GA3OX1* and *GA3OX2* expression in 3‐wk‐old *swp73a* seedlings, and the presence of SWP73A at the *GA3OX* loci, we investigated whether *swp73a* influences germination response after stratification, a stage when *GA3OX1* and *GA3OX2* expression naturally peaks (Fig. S13). Germination of the *swp73a* mutant was accelerated compared with that of WT (Fig. 6a), suggesting that SWP73A modulates the germination rate.

We used complemented SWP73A::SWP73A‐GFP transgenic lines in the *swp73a* background exhibiting reversed *swp73a* germination phenotype. In *swp73a* mutant seedlings, *GA3OX1* and *GA3OX2* reached expression peaks faster than in WT plants. Their expression was altered in *swp73a* plants before the radicle emerged from the seed coat, while after 48 h of germination, their levels resembled those in WT plants (Fig. 6b,c). The *GA3OX3* expression is not activated during imbibition and was not altered in *swp73a* plants. ChIP‐qPCR analysis in dry seeds showed four‐fold enrichment of SWP73A‐GFP on *GA3OX1* and two‐fold enrichment on *GA3OX3* gene body regions (Fig. 6d,e). After 24 h of imbibition, SWP73A was recruited to the *GA3OX2* locus and was not present on the *GA3OX3* locus (Fig. 6f). The SWP73A protein occupied the 5′UTR of *GA3OX1* and *GA3OX2* loci. After 48 h of imbibition, SWP73A was no longer detectable on *GA3OX* genes (Fig. 6g). In line with chromatin occupancy results, we found a widespread signal of SWP73A‐GFP protein in embryo cells in dry seeds and after 24 h of imbibition. However, after 48 h, the GFP signal was no longer detectable (Fig. 6h). Since SWP73A is a subunit of the BAS‐A SWI/SNF complex, we asked whether its absence influences chromatin marks. The enrichment for H3K9me2 on *GA3OX1/GA3OX2* loci was decreased about two‐ to three‐fold in the *swp73a* background, while H3K4me3 enrichment was about two‐fold higher in the absence of SWP73A (Fig. 6i,j). Taken together, our data indicate a pivotal role of the SWP73A‐containing BAS‐A subclass of SWI/SNF CRCs in orchestrating germination dynamics in Arabidopsis through the modulation of the expression of *GA3OX1* and *GA3OX2*.

### SWP73A and SWP73B play different roles in metabolic regulation

**Fig. 7:**
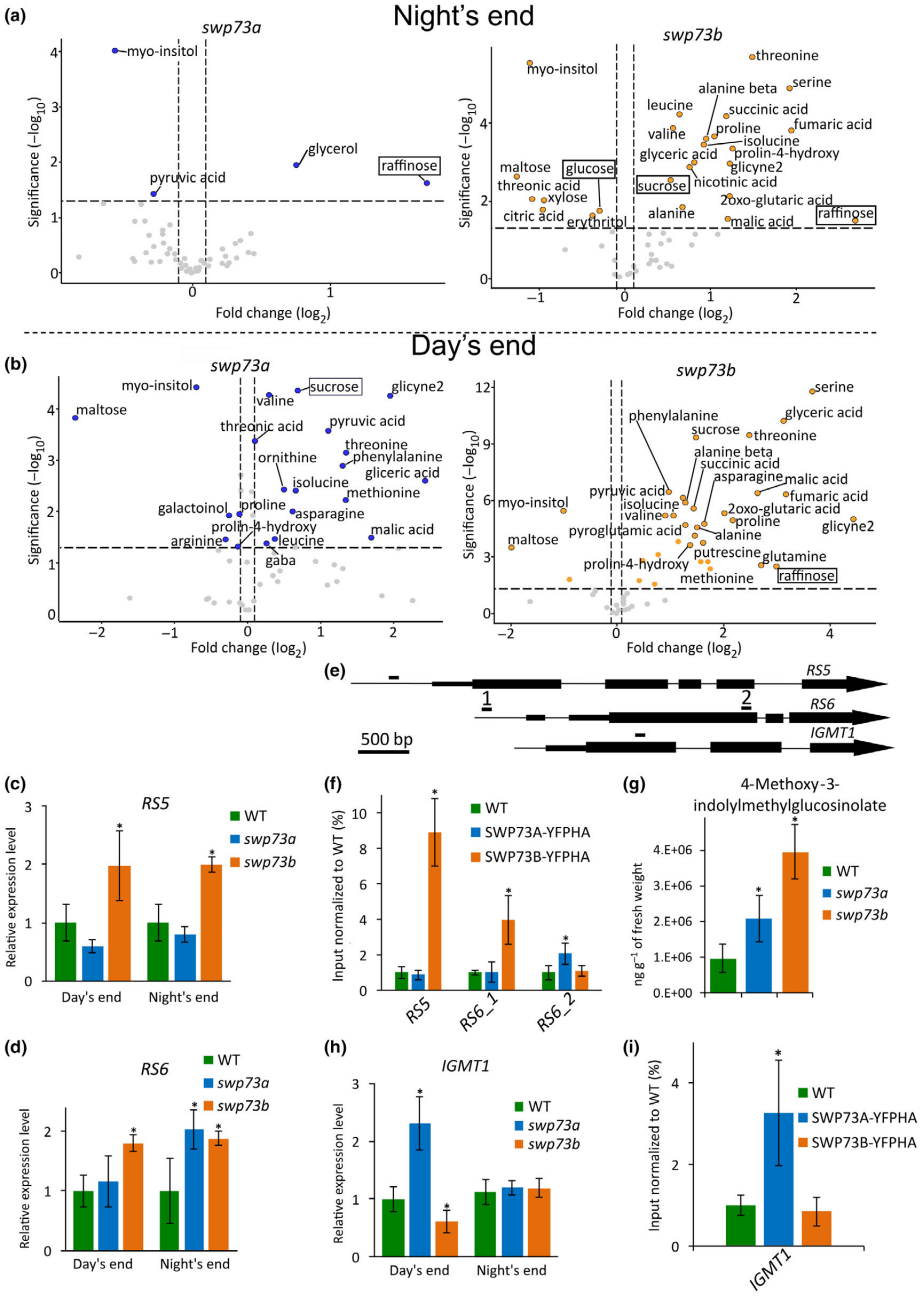
*swp73a* and *swp73b* mutations have a different effect on metabolome profiles in Arabidopsis. (a) Quantity of primary metabolites in the *swp73a* and *swp73b* mutants collected at night's end. (b) Quantity of primary metabolites in the *swp73a* and *swp73b* mutants collected at the end of the day. Relative expression of *RAFFINOSE SYNTHASE 5* (c) and *RAFFINOSE SYNTHASE 6* (d) in *swp73a* and *swp73b*. Error bars denote the SD, while asterisks indicate statistical significance (*n* = 3, *P*‐value < 0.05, Student's *t*‐test). (e) Scheme of the *RS5*, *RS6* and *IGMT1* genes with amplicon positions corresponding to (f, i). (f) SWP73A and SWP73B bind to the regulatory region of *RS5* and *RS6* (*n* = 3, *P*‐value < 0.05, Student's *t*‐test). (g) 4‐Methoxy‐3‐indolylmethylglucosinolate amount in *swp73a* and *swp73b* collected at the end of the day. Error bars denote the SD, while asterisks indicate statistical significance (*n* = 3, *P*‐value < 0.05, Student’s *t*‐test). (h) Relative expression of *INDOLE GLUCOSINOLATE O‐METHYLTRANSFERASE 1* in *swp73a* and *swp73b.* Error bars denote the SD, while asterisks indicate statistical significance (*n* = 3, *P*‐value < 0.05, Student's *t*‐test). (i) SWP73A binds to the *IGMT1* locus, as shown by chromatin immunoprecipitation (ChIP)‐qPCR analysis. Error bars denote the SD, while asterisks indicate statistical significance (*n* = 3, *P*‐value < 0.05, Student's *t*‐test).

Transcriptomic changes caused by *swp73* mutations and the genome‐wide distribution of SWP73A/B proteins suggest that various classes of SWI/SNF complexes may be involved in the control of metabolism‐related processes. We found that both *swp73a* and *swp73b* plants were able to germinate on sucrose‐free medium; however, *swp73b* plants exhibited enhanced growth retardation, and their length at Day 7 did not exceed 2 mm (Fig. S14a,b). *swp73a* plants grown in the absence of sugar resembled WT plants. We investigated the metabolic profile for 52 metabolites and found that at the end of the night, the levels of *myo*‐inositol, pyruvic acid, glycerol and raffinose were altered in the *swp73a* mutant (Fig. 7a; Table S28).

Both *swp73* mutants overaccumulated sucrose at the end of the day (Fig. 7b; Table S28). The accumulation of raffinose in the *swp73b* mutant occurred irrespective of the time of day, while glucose was accumulated only at the end of the night.

The *swp73a* metabolome profile changed dramatically at the end of the day (Fig. 7b). The number of affected metabolites increased to 25, exceeding even the number of metabolites affected in the *swp73b* mutant line, where 23 metabolites were altered. The *swp73a* mutation resulted in amino acid overaccumulation; for example, increases were noted in serine, threonine and proline levels. Their accumulation was in line with stress response genes activated in *swp73a*, as shown in our RNA‐seq data (Tables S3, S14, S15).

SWP73A directly regulates the expression of *RAFFINOSE SYNTHASE 6* (*RS6/Dark Inducible 10*/*DIN10*), whereas SWP73B targets *RAFFINOSE SYNTHASE 5* (*RS5*) (Tables S10, S11). The striking upregulation of *RS6* in the *swp73a*/*SWP73A*; *swp73b*/*swp73b* sesquimutants suggests that both SWP73s may control *RS6* expression (Table S8). We found that *RS5* exhibited elevated expression only in the *swp73b* mutant, while *RS6* was upregulated in the *swp73a* mutant at the end of the night and constantly in the *swp73b* mutant (Fig. 7c,d). Confirmatory ChIP‐qPCR analysis indicated that both SWP73 directly regulate *RS6* expression and SWP73B binds the *RS5* promoter (−100 bp to TSS) (Fig. 7e,f). SWP73B bound the promoter region of the *RS6* (−400 bp to TSS) gene while SWP73A targeted the *RS6* gene body (+2000 bp from TSS), suggesting that subclasses of SWI/SNF complexes carrying SWP73A or SWP73B are involved in the direct control of *RS6* gene expression.

In line with the raffinose overaccumulation, we detected alterations in the expression of glucosinolate metabolism genes in the *swp73a* mutant. Glucosinolates are important for the defence responses against biotic stresses, and SWP73A has a significant role in the immune response in Arabidopsis. *swp73a* accumulated 4‐methoxy‐3‐indolylmethylglucosinolate (4mIMG) (Fig. 7g), which is induced by SA (Mikkelsen *et al*., 2003), and upregulated indole glucosinolate O‐methyltransferase 1 (*IGMT1*) at the day's end (Fig. 7h). The *swp73b* accumulated 4‐metindolyl methyl glucosinolateinolate and exhibited elevated levels of *IGMT3* and *IGMT5*, but not *IGMT1* (Table S6). Using ChIP‐qPCR, we showed that SWP73A targeted the gene body region (+500 bp from TSS) of *IGMT1* at the end of the day (Fig. 7i). These data indicate that SWI/SNF BAS‐A complexes containing SWP73A and complexes carrying SWP73B may differentially regulate metabolic processes to fine‐tune homeostasis.

## Discussion

SWI/SNF CRCs are evolutionarily conserved. However, in higher eukaryotes, multiplication of genes encoding some of their subunits occurred, correlating with a diversification of their functions. The subfamily of SWI/SNF CRCs consists of three subclasses: in humans (cBAF‐canonical, pBAF‐polybromo and ncBAF‐noncanonical) and in Arabidopsis, respectively: SAS, MAS and BAS (Mashtalir *et al*., 2018; J. Guo *et al*., 2022). Such classification is obviously not fully robust due to the potential tissue/developmental specialisation of certain subunits, their combinatorial assembly and evidences from genetic and protein–protein interaction studies. Pairwise mutation of some SWI/SNF subunits in humans and Arabidopsis causes synthetic lethality (Bezhani *et al*., 2007; Sang *et al*., 2012; Michel *et al*., 2018). The SWP73/BAF60 family is one of the best examples of functionally specialised SWI/SNF subunits, which are multiplied in dicots and mammals. In humans, BAF60 subunits exhibit tissue‐specific functions, and they are involved in SWI/SNF CRCs assembly (Wang *et al*., 2018). The phenotypic alterations presented by single Arabidopsis mutants (Sacharowski *et al*., 2015) and the synthetic lethality occurring during embryonic development when both SWP73A and SWP73B are lost indicate that they exhibit not only shared but also unique functions.

**Fig. 8:**
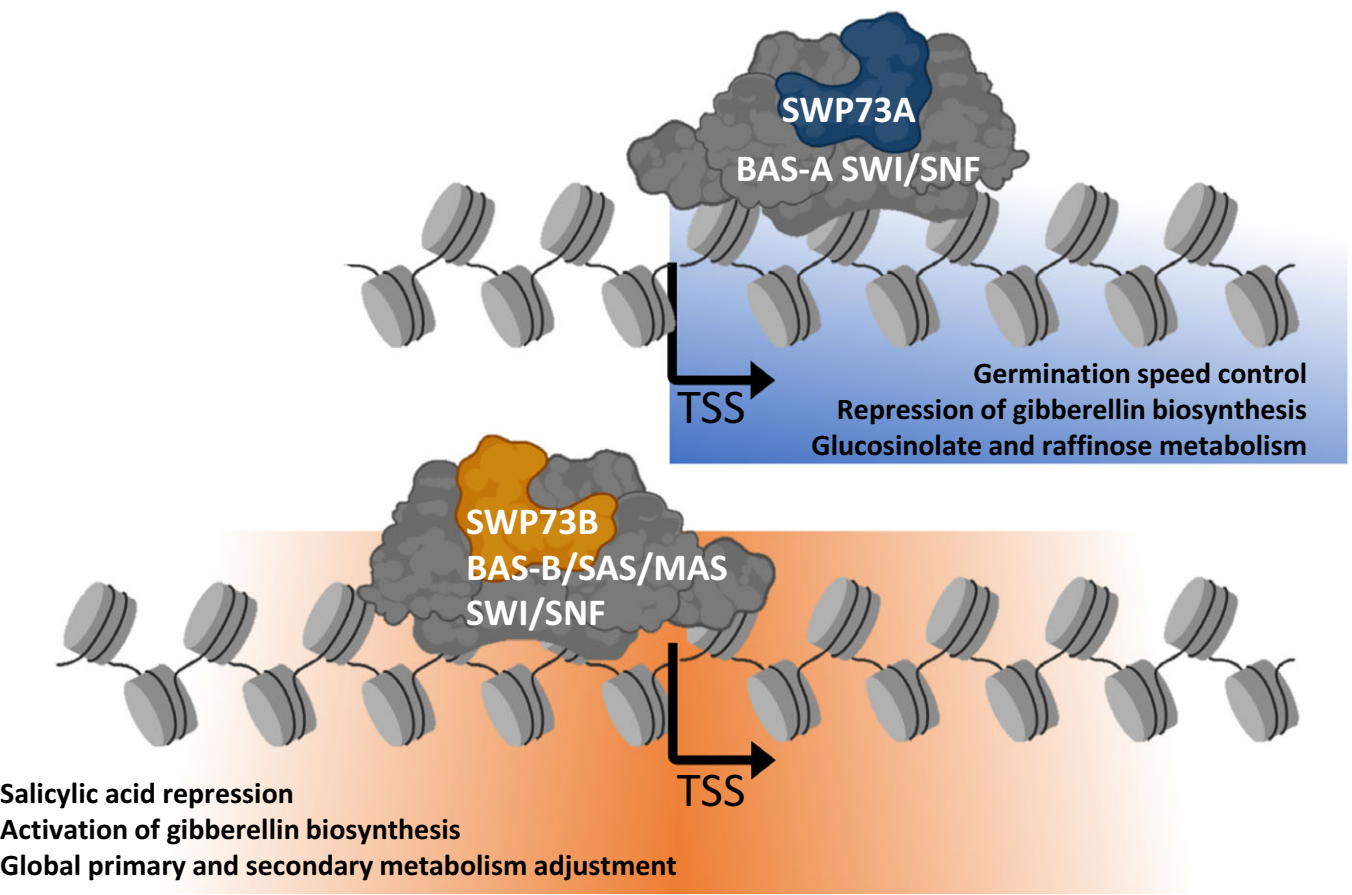
Hypothetical working model summarising regulatory functions of Arabidopsis switch‐defective/sucrose nonfermentable (SWI/SNF) complex subclasses containing SWP73A and SWP73B. SWP73A can form a subclass of BRM‐associated SWI/SNF (BAS)‐A BRM‐containing SWI/SNF complex and binds to gene body regions. SWP73A controls genes involved in auxin biosynthesis and response. Its loss of function leads to decreased auxin levels and affected lateral root formation upon auxin treatment. It represses the expression of raffinose and glucosinolate biosynthesis genes and negatively modulates germination dynamics. However, SWP73B has broader functions than SWP73A. SWP73B forms the BAS‐B subclass of BRM‐SWI/SNF chromatin remodelling complexes (CRCs), and it is indispensable in the formation of MINUSCULE‐associated SWI/SNF (MAS)‐ and SYD‐associated SWI/SNF (SAS)‐SWI/SNF complexes. SWP73B‐containing SWI/SNF subclasses mainly occupy promoter and 5'UTR regions. *swp73b* mutation leads to decreased gibberellin levels and overaccumulation of salicylic acid. SWP73B affects metabolism control, including raffinose and glucosinolates. Created in BioRender (https://BioRender.com/z26m589).

Here, we identified so‐far nonrecognised functions of the SWP73A and SWP73B subunits. We found that SWP73A is present only in a specific subclass of the BAS complex. We, therefore, named this subclass BAS‐A. By contrast, SWP73B, in addition to MAS and SAS subclasses of SWI/SNF CRCs, occurs in the BAS‐B subclass. Consistently, with the results of our previous study, we demonstrate that the SWP73A and SWP73B subunits exhibit only partially redundant functions. The regulatory role is primarily performed by the SWP73B subunit, while SWP73A functions specifically in the control of such processes as repression of gibberellin biosynthesis during germination, SA biosynthesis at night, auxin biosynthesis and response, and metabolic control (Fig. 8). The SWP73A protein mainly targets gene bodies, while SWP73B is recruited to promoter regions and regions corresponding to 3′ UTR, resembling the situation in humans where BAF60a (SWP73‐type) also occupies gene bodies, not being present in the 5′ UTR and proximal promoter regions (Alajem *et al*., 2015).

The function of SWP73A is more clearly noticeable when the SWP73B protein is absent, as demonstrated by the fact that transcriptomic and phenotypic changes are more pronounced in the *swp73a*/*SWP73A*; *swp73b*/*swp73b* sesquimutant. Our proteomic results may explain the phenotypic differences caused by the *swp73a* and *swp73b* mutations. Minor changes in the *swp73a* mutant's phenotype result from the loss of SWP73A‐containing BAS‐A subclass and are likely compensated by alternative BAS complexes containing SWP73B (BAS‐B). By contrast, drastic effects of the *swp73b* mutation reflect a loss of function of BAS‐B, MAS and SAS subclasses of the SWI/SNF complexes, as *swp73b* plants carry only SWP73A‐containing BAS‐A complexes. This suggests that a single BAS‐A complex may suffice for viability. However, when one *SWP73A* allele is inactive in *swp73a*/*SWP73A*; *swp73b*/*swp73b* sesquimutant plants, severe defects exacerbate, thereby reducing the functionality of the remaining BAS‐A class. This finding is clearly supported by the increasing number of DEGs observed in *swp73a*/*SWP73A*; *swp73b*/*swp73b* sesquimutant plants in comparison with the single *swp73a* and *swp73b* mutant lines, and by the inconsistency between the number of DEGs and direct targeting of SWP73A and SWP73B to their loci. Such phenomenon may likely be explained by the occupancy profiles of SWP73A and SWP73B, as both target intergenic regions. Thus, the subclasses of SWI/SNF CRCs carrying these subunits could be responsible for the occupancy of distal regulatory sequences or involved in the formation of higher order chromatin structures, as shown by Jégu *et al*. (2017). Alternatively, altered genome‐wide distribution of remaining subclasses of SWI/SNF in the *swp73a* or *swp73b* mutant lines might also explain this effect.

We found that the *swp73b* mutation affects the presence of the SWI3B subunit on *ICS1*, *ICS2* and *MES9* loci involved in SA biosynthesis. A similar situation occurred in the case of *GA2OX1*, *GA2OX2*, *GA20OX2* and *GA3OX2* promoter regions of genes involved in GA biosynthesis, where SWI3B targeting was abolished by the *swp73b* mutation. These results indicate the involvement of the MAS complex in the control of these loci. By contrast, the SWI3B presence on the *GA3OX3* promoter region was not abolished by the *swp73b* mutation, indicating the presence of an atypical MAS complex without SWP73B on this locus in the *swp73b* mutant line. Conversely, we found the SWI3B presence on the *GA3OX2* and *GA3OX3* gene bodies; however, these regions are targeted by SWP73A but not by SWP73B, and the absence of either SWP73A or SWP73B did not affect SWI3B binding. Such a phenomenon may be explained by the formation of atypical subclasses of SWI/SNF CRCs other than reported so far when some subunits are missing in the mutants; for example, atypical MAS complexes carrying SWP73A. An alternative explanation is that in the absence of functional BAS‐A complexes in the *swp73a* line, the genomic localisation of MAS complexes carrying SWP73B is altered, and they tend to occupy the target sequences in *swp73a* as both SWP73 undergo ubiquitous expression. The existence of atypical subclasses of SWI/SNF CRCs other than BAS‐A, BAS‐B, SAS and MAS is additionally supported by our intriguing finding that SWP73A and B subunits interact and may occur with low frequency in the same SWI/SNF complexes. This is in line with the observation for the other SWI/SNF subunits: BRIP1 and BRIP2 (Yu *et al*., 2020). Moreover, Li *et al*. (2016) identified two central catalytic subunits of SWI/SNF (SYD and BRM) precipitating jointly. In mammals, Mashtalir *et al*. (2018) identified Baf60b and Baf60c precipitating with Baf60a, although structurally they are mutually exclusive subunits of SWI/SNF (He *et al*., 2021). Our data showing that only 1 out of 250 interactions was identified as a direct interaction between SWP73A and SWP73B underscore the rarity of their co‐occurrence in the same complexes. This suggests that, although they can interact, their primary functions are likely executed as distinct complexes, resulting in unique regulatory outcomes. We propose that further investigation of the specific conditions under which these interactions occur could provide insights into their functional interplay. For example, the presence of SWP73A may enhance the stability or functionality of certain SWP73B‐containing complexes, or vice versa, under specific developmental or environmental contexts. We postulate that the existence of other atypical subclasses of SWI/SNF CRCs may be a general paradigm, and given the frequent mutation of SWI/SNF subunits in cancer and other human diseases, further examination of the existence of such atypical (or atypically distributed) subclasses when some SWI/SNF subunits are absent is a vital path of further research in Arabidopsis and humans.

Significant enrichment for genes involved in response to hormones was found as the common and specific targets of SWP73A and SWP73B proteins. We found genes involved in hormonal signalling/biosynthesis pathways, for example, auxin, cytokinin, gibberellin and SA. Thus, to further characterise the interplay between SWP73A and SWP73B, we performed standard phenotypic tests using various phytohormones and treatments. We found the differential effects of *swp73a* and *swp73b* mutations on hormone biosynthesis and hormone content in Arabidopsis, with a clearly broader function of SWP73B than of SWP73A. However, the increased number of lateral roots of *swp73a* upon auxin treatment, the presence of SWP73A in root tips and lateral root primordia, and the alterations in its localisation in the root upon auxin treatment, decreased indole‐3‐acetic level alongside the misregulation of auxin transport genes, such as *PIN1*, *PIN4* and *GH3.17*, in the *swp73a* mutant indicate that the SWP73A‐containing BAS‐A subclass may directly fine‐tune auxin homeostasis in a different way than SWP73B, as the *swp73b* mutation does not affect the bioactive auxin level, although it exhibits auxin hypersensitivity and auxin‐related phenotypic traits. These findings, together with the pronounced role of SWP73A during germination, indicate that the function of BAS‐A, the subclass of SWP73A‐containing SWI/SNF complexes absent in the *swp73a* line, cannot be fully replaced by SWP73B‐containing classes of SWI/SNF complexes.

The SWI/SNF CRCs containing SWP73B are not the only factors controlling the hormonal pathways, as there is a lot of information indicating the involvement of subunits of other CRCs, lncRNA and histone deacetylases. The relationship between the SWI/SNF CRCs and histone modifications is characterised by complex interactions that function both upstream and downstream of chromatin remodelling. Histone acetylation can enhance the recruitment of SWI/SNF complexes through interactions with histone acetyltransferases (HATs), such as p300 and CBP, which acetylate histones and facilitate SWI/SNF binding (Ogiwara *et al*., 2011). Conversely, SWI/SNF remodelling activity can also influence histone acetylation patterns by exposing nucleosome‐bound DNA, allowing for further histone modifications (Ferreira *et al*., 2011; Ogiwara *et al*., 2011). Consistently, we found a differential effect of the loss of SWP73A or SWP73B on particular histone modification deposition, further supporting the role of various subclasses of SWI/SNF CRCs in additional layers of gene expression control, thus representing an attractive direction of future study. Our study broadens the knowledge about chromatin‐associated repressors of SA biosynthesis. SA and gibberellins can positively modulate each other (Alonso‐Ramírez *et al*., 2009; Emamverdian *et al*., 2020), and we showed that SWP73B is required for synergistic crosstalk between them. We show here the complementary role of the SWI/SNF complexes in GA biosynthesis executed by SWP73 subunits. They coordinate the expression of *GA3OX* genes crucial for bioactive gibberellin biosynthesis. Knock‐out of *SWP73B*, similarly to *BRM* and *SWI3C*, results in a decreased level of gibberellin, showing all subunits of the BAS‐SWI/SNF complex are required for gibberellin biosynthesis (Archacki *et al*., 2013; Sarnowska *et al*., 2013). We also observed higher expression of *GID1B* in the *swi3b‐3* and *swi3c‐1* mutants (Sarnowska *et al*., 2013, 2023), suggesting that MAS and BAS‐B classes may cooperate to repress *GID1B*.

Substantial metabolite alterations and genome‐wide data concerning SWP73A/B showed that both SWP73s could cooperatively and separately influence metabolism. *SWP73A* knock‐out caused the enrichment of glucosinolate, organic acid, alpha‐amino acid and secondary metabolic GO process. The knock‐out of *SWP73B* resulted in altered expression of multiple genes related to metabolic processes (e.g. Qua Quine Starch, Senescence‐Associated and QQS‐Related, *S*‐adenosyl‐L‐methionine methyltransferase, galactose oxidase, glutathione *S*‐transferases). Interestingly, the phenotype alterations in *swp73b* were enhanced when seedlings were cultivated on a medium without any sugar, supporting an essential role of SWP73B in sugar sensing and/or metabolism regulation. SWI/SNF is inextricably linked with metabolism control from yeast to plants and mammals, as indicated by the ChIP‐seq results showing occupancy of SWI/SNF subunits on genes involved in amino acid and sugar metabolism (Euskirchen *et al*., 2011). We identified the diversity of SWP73A/B involvement in metabolic processes analogous to the mammalian BAF60 subunits, where BAF60a and BAF60c regulate metabolism in the liver and skeletal muscle (Wang *et al*., 2018). BAF60a also integrates the circadian clock with metabolism (Tao *et al*., 2011) in mice, while in Arabidopsis, both SWP73 link these two processes.

Our studies, together with other reports, indicate that the interdependence between SWP73/BAF60 is an evolutionarily conserved feature. Functional differentiation of SWI/SNF complex classes in transcriptional regulation is likely a primary mechanism that may have been established before the Kingdoms' divergence. Our results thus broaden the picture of the regulatory network involved in fine‐tuning hormone crosstalk, metabolism, germination and other important regulatory processes.

## Competing interests

None declared.

## Author contributions

SPS performed most of the experimental work, data analyses and writing. TJS planned experiments, supervised work, analysed data and wrote the article. DG‐Z and PO performed phenotype tests. BH performed RNA‐seq. JS, MC, KN, EB and ATR created constructs and complemented SWI3D‐YFPHA and BSH‐YFPHA lines. JS performed BSH‐IP. DLZ and SA performed metabolic analysis. TT and PG performed metabolomic data analysis. M‐RH performed hormone measurements. AH and SCS performed mass‐spec analysis. SCS and CK performed mass‐spec analysis. RF performed scanning electron microscopy. PC performed BiFC. SK performed confocal scanning and participated in germination assays. EG, HN, ARF and CK analysed the data and edited the article.

## Disclaimer

The New Phytologist Foundation remains neutral with regard to jurisdictional claims in maps and in any institutional affiliations.

## Supporting information


**Fig. S1** SWP73A and SWP73B in Arabidopsis have different impacts on transcriptome and various profiles of genome‐wide distribution.
**Fig. S2** The phenotype of SWP73A‐ and SWP73B‐complemented Arabidopsis lines.
**Fig. S3** The analysis of SWP73A, SWP73B, SYD and BRM occupancy at specific and co‐occupied gene sets in Arabidopsis.
**Fig. S4** SWP73A/SWP73B co‐targeted genes and chromatin landscapes of BAS‐A, BAS‐B and SAS.
**Fig. S5** Arabidopsis SWP73A and SWP73B directly interact.
**Fig. S6** SWP73A and SWP73B bind to genes involved in multiple hormonal pathways in Arabidopsis.
**Fig. S7** *swp73a* and *swp73b* Arabidopsis mutants features related to auxin biosynthesis and response.
**Fig. S8** Auxin‐induced expression and ChIP‐seq analysis of BAS‐A targets in WT and *swp73a* Arabidopsis plants.
**Fig. S9** BAP treatment and IP profiling in WT, *swp73a* and *swp73b* indicate differential roles of Arabidopsis SWP73A and SWP73B in cytokinin signalling.
**Fig. S10** Gibberellin treatment partially reverses *swp73b* developmental defects.
**Fig. S11** Salicylic acid signal transduction is affected in *swp73* Arabidopsis mutant lines.
**Fig. S12** SWP73 does not control the expression of genes involved in the initial steps of gibberellin biosynthesis in Arabidopsis.
**Fig. S13** Expression patterns of Arabidopsis *SWP73A*, *SWP73B*, *GA3OX1*, *GA3OX2* and *GA3OX3* genes presented in EFP public databases during germination.
**Fig. S14** *swp73a* and *swp73b* exhibit differential Arabidopsis response to the presence of sucrose in the medium.

**Table S1** Primers list.
**Table S2** Genes that exhibit significantly different expression in swp73a/SWP73A Arabidopsis plants compared with WT (FDR < 0.05).
**Table S3** Genes that exhibit significantly different expression in *swp73a* Arabidopsis mutant compared with WT (FDR < 0.05).
**Table S4** Genes that exhibit significantly different expression in *swp73b* Arabidopsis mutant compared with WT (FDR < 0.05).
**Table S5** Genes that exhibit significantly different expression in *swp73a*/*SWP73A*; *swp73b*/*swp73b* Arabidopsis sesquimutant compared with WT (FDR < 0.05).
**Table S6** GO categories enriched in *swp73b* upregulated genes.
**Table S7** GO categories enriched in *swp73b* downregulated genes.
**Table S8** GO categories enriched in *swp73a*/*SWP73A*; *swp73b*/*swp73b* upregulated genes.
**Table S9** GO categories enriched in *swp73a*/*SWP73A*; *swp73b*/*swp73b* downregulated genes.
**Table S10** SWP73A peaks identified by ChIP‐seq.
**Table S11** SWP73B peaks identified by ChIP‐seq.
**Table S12** List of genes occupied by SWP73A or SWP73B in Arabidopsis seedlings.
**Table S13** List of genes with changed expression in Arabidopsis *swp73a*/*SWP73A*; *swp73b*/*swp73b* and occupied by SWP73A and/or SWP73B.
**Table S14** GO categories enriched in *swp73a*/*SWP73A*; *swp73b*/*swp73b* upregulated genes and targeted by SWP73A.
**Table S15** GO categories enriched in *swp73a*/*SWP73A*; *swp73b*/*swp73b* downregulated genes and targeted by SWP73A.
**Table S16** GO categories enriched in *swp73a*/*SWP73A*; *swp73b*/*swp73b* upregulated genes and targeted by SWP73B.
**Table S17** GO categories enriched in *swp73a*/*SWP73A*; *swp73b*/*swp73b* downregulated genes and targeted by SWP73B.
**Table S18** GO categories enriched in *swp73a*/*SWP73A*; *swp73b*/*swp73b* upregulated genes and targeted by both SWP73s.
**Table S19** GO categories enriched in *swp73a*/*SWP73A*; *swp73b*/*swp73b* downregulated genes and targeted by both SWP73s.
**Table S20** Arabidopsis SWP73A LC‐MS/MS data.
**Table S21** Arabidopsis SWP73B LC‐MS/MS data.
**Table S22** Arabidopsis SWI3D LC‐MS/MS data.
**Table S23** Arabidopsis BSH LC‐MS/MS data.
**Table S24** List of genes occupied by BRM or SYD in Arabidopsis seedlings.
**Table S25** Comparison of genes, *hormone‐related* genes and targeted by SWP73A or SWP73B in Arabidopsis.
**Table S26** GO categories enriched in *hormone‐related* genes and targeted by SWP73A in Arabidopsis.
**Table S27** GO categories enriched in *hormone‐related* genes and targeted by SWP73A in Arabidopsis.
**Table S28** Metabolite annotation and documentation for GC‐ and LC‐MS data.
Please note: Wiley is not responsible for the content or functionality of any Supporting Information supplied by the authors. Any queries (other than missing material) should be directed to the *New Phytologist* Central Office.

## Data Availability

RNA‐seq data are available in the GEO database under accession no.: GSE261097. The mass spectrometry proteomics data have been deposited to the ProteomeXchange Consortium via the PRIDE partner repository with the dataset identifier PXD056187. Sequence data from this article can be found in the GenBank/EMBL databases under accession nos.: SWP73A (AT3G01890), SWP73B (AT5G14170), SWI3A (AT2G47620), SWI3B (AT2G33610), SWI3C (AT1G21700), SWI3D (AT4G34430) and BSH (AT3G17590). The cartoon in Fig. 8 was created in BioRender (https://BioRender.com/z26m589).
