# Supplementary material for: BAF60/SWP73 subunits define subclasses of SWI/SNF chromatin remodelling complexes in Arabidopsis
Source: New Phytol. 2025 May 22;247(2):791–812. doi: 10.1111/nph.70182 (PMC12177292; doi:10.1111/nph.70182)
Supplement: Supplementary file 1 — Fig. S1 SWP73A and SWP73B in Arabidopsis have different impacts on transcriptome and various profiles of genome‐wide distribution. Fig. S2 The phenotype of SWP73A‐ and SWP73B‐complemented Arabidopsis lines. Fig. S3 The analysis of SWP73A, SWP73B, SYD and BRM occupancy at specific and co‐occupied gene sets in Arabidopsis. Fig. S4 SWP73A/SWP73B co‐targeted genes and chromatin landscapes of BAS‐A, BAS‐B and SAS. Fig. S5 Arabidopsis SWP73A and SWP73B directly interact. Fig. S6 SWP73A and SWP73B bind to genes involved in multiple hormonal pathways in Arabidopsis. Fig. S7 swp73a and swp73b Arabidopsis mutants features related to auxin biosynthesis and response. Fig. S8 Auxin‐induced expression and ChIP‐seq analysis of BAS‐A targets in WT and swp73a Arabidopsis plants. Fig. S9 BAP treatment and IP profiling in WT, swp73a and swp73b indicate differential roles of Arabidopsis SWP73A and SWP73B in cytokinin signalling. Fig. S10 Gibberellin treatment partially reverses swp73b developmental defects. Fig. S11 Salicylic acid signal transduction is affected in swp73 Arabidopsis mutant lines. Fig. S12 SWP73 does not control the expression of genes involved in the initial steps of gibberellin biosynthesis in Arabidopsis. Fig. S13 Expression patterns of Arabidopsis SWP73A, SWP73B, GA3OX1, GA3OX2 and GA3OX3 genes presented in EFP public databases during germination. Fig. S14 swp73a and swp73b exhibit differential Arabidopsis response to the presence of sucrose in the medium. [file NPH-247-791-s001.pdf]

***New Phytologist* Supporting Information**

Article title: BAF60/SWP73 Subunits Define Subclasses of SWI/SNF Chromatin Remodelling Complexes in Arabidopsis

Authors: **Sebastian P. Sacharowski, Szymon Kubala, Pawel Cwiek, Jaroslaw Steciuk, Dominika Gratkowska-Zmuda, Paulina Oksinska, Ernest Bucior, Anna T. Rolicka, Monika Ciesla, Klaudia Nowicka, Saleh Alseekh, Takayuki Tohge, Patrick Giavalisco, Dorota L. Zugaj, Sara C. Stolze, Anne Harzen, Rainer Franzen, Bruno Huettel, Elzbieta Grzesiuk, Mohammad-Reza Hajirezaei, Hirofumi Nakagami, Csaba Koncz, Alisdair R. Fernie, Tomasz J. Sarnowski**

Article acceptance date: 07 April 2025

The following Supporting Information is available for this article:

Fig. S1 SWP73A and SWP73B in Arabidopsis have different impacts on transcriptome and various profiles of genome-wide distribution.

Fig. S2 The phenotype of SWP73A- and SWP73B-complemented Arabidopsis lines.

Fig. S3 The analysis of SWP73A, SWP73B, SYD, and BRM occupancy at specific and co-occupied gene sets in Arabidopsis.

Fig. S4 SWP73A/SWP73B co-targeted genes and chromatin landscapes of BAS-A, BAS-B, and SAS.

Fig. S5 Arabidopsis SWP73A and SWP73B directly interact.

Fig. S6 SWP73A and SWP73B bind to genes involved in multiple hormonal pathways in Arabidopsis.

Fig. S7 *swp73a* and *swp73b* Arabidopsis mutants features related to auxin biosynthesis and response.

Fig. S8 Auxin-Induced expression and ChIP-seq analysis of BAS-A targets in WT and *swp73a* Arabidopsis plants.

Fig. S9 BAP treatment and IP profiling in WT, *swp73a*, and *swp73b* indicate differential roles of Arabidopsis SWP73A and SWP73B in cytokinin signalling.

Fig. S10 Gibberellin treatment partially reverses *swp73b* developmental defects.

Fig. S11 Salicylic acid signal transduction is affected in *swp73* Arabidopsis mutant lines.

Fig. S12 SWP73 do not control the expression of genes involved in the initial steps of gibberellin biosynthesis in Arabidopsis.

Fig. S13 Expression patterns of Arabidopsis *SWP73A, SWP73B, GA3OX1, GA3OX2* and *GA3OX3* genes presented in EFP public databases during germination.

Fig. S14 *swp73a* and *swp73b* exhibit differential Arabidopsis response to the presence of sucrose in the medium.

(a)

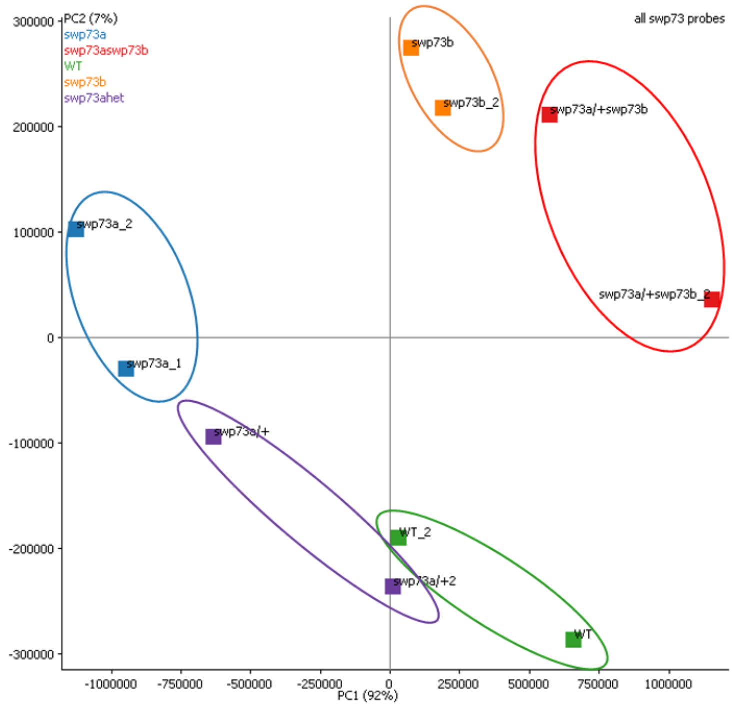

(b)

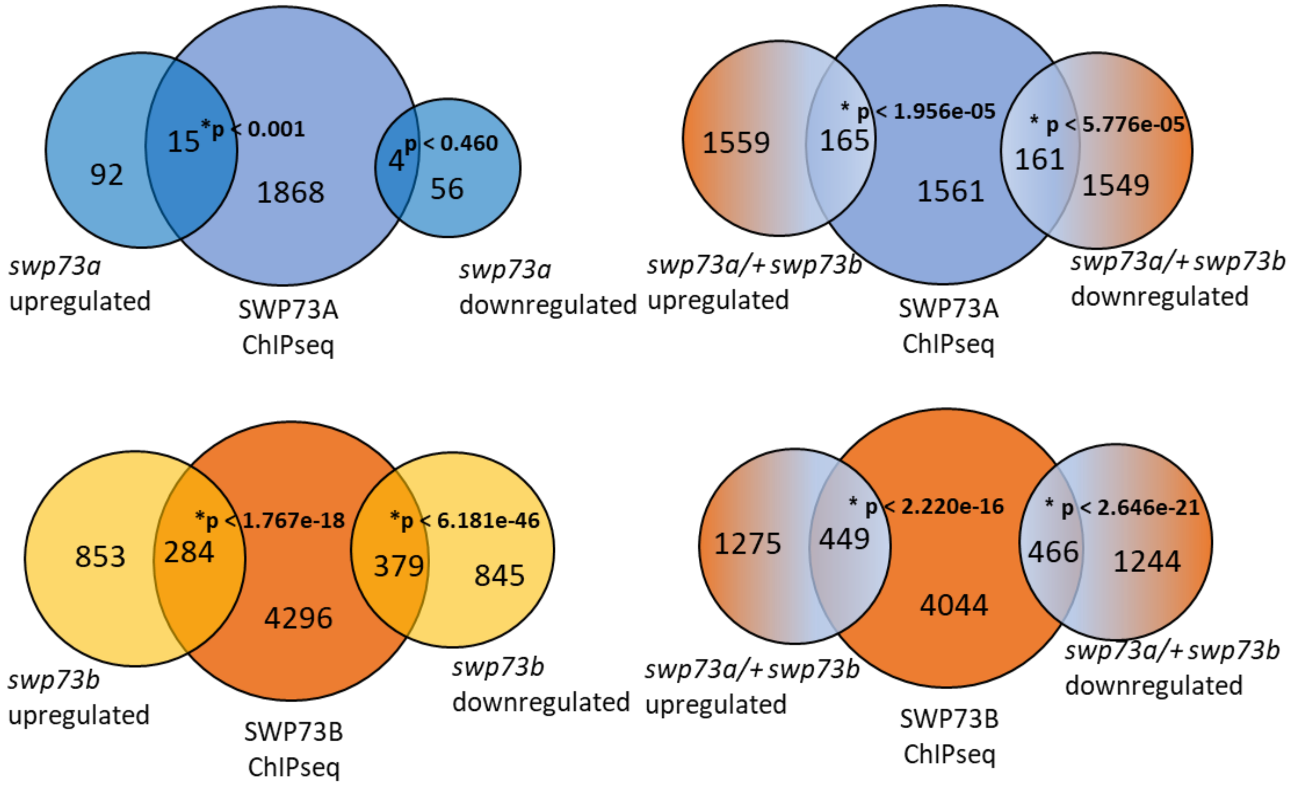

Fig. S1 SWP73A and SWP73Bin Arabidopsis have different impacts on transcriptome and various profiles of genome-wide distribution. (a) PCA plot of biological replicates included in RNA-Seq analysis based on genes altered in *swp73a* and *swp73b.* **(b)** SWP73A and SWP73B exhibit differential genome-wide distribution and various direct and indirect effects on transcriptome, which is enhanced in

swp73a/*SWP73A*; *swp73b*/*swp73b* sesquimutant plants. *P* values were determined by hypergeometric test.

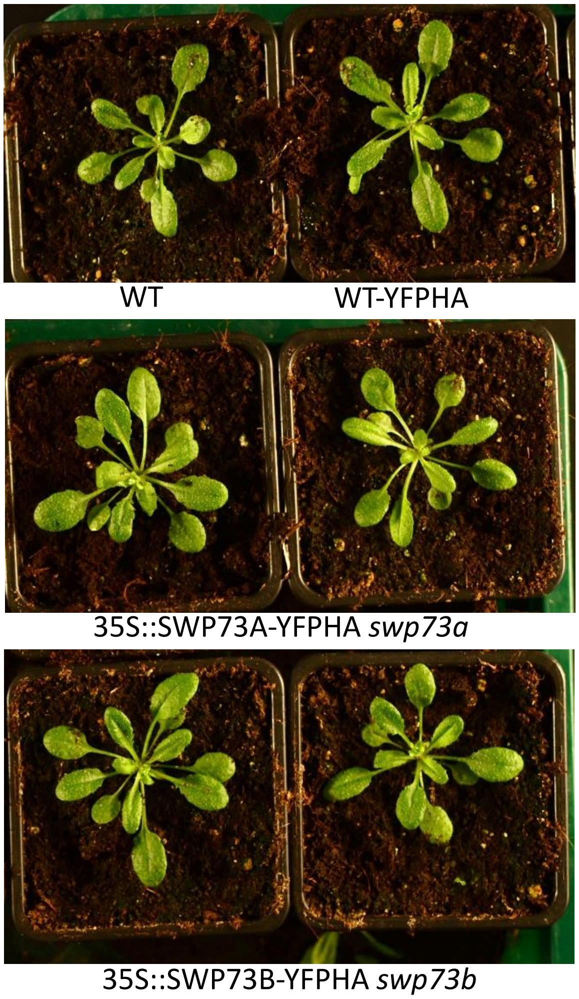

Fig. S2 The phenotype of SWP73A- and SWP73B-complemented Arabidopsis lines. The phenotype of 35S::YFP-HA, 35S::SWP73A-YFP-HA *swp73a*, and 35S::SWP73B-YFP-HA *swp73b* Arabidopsis lines exhibit WT characteristics.

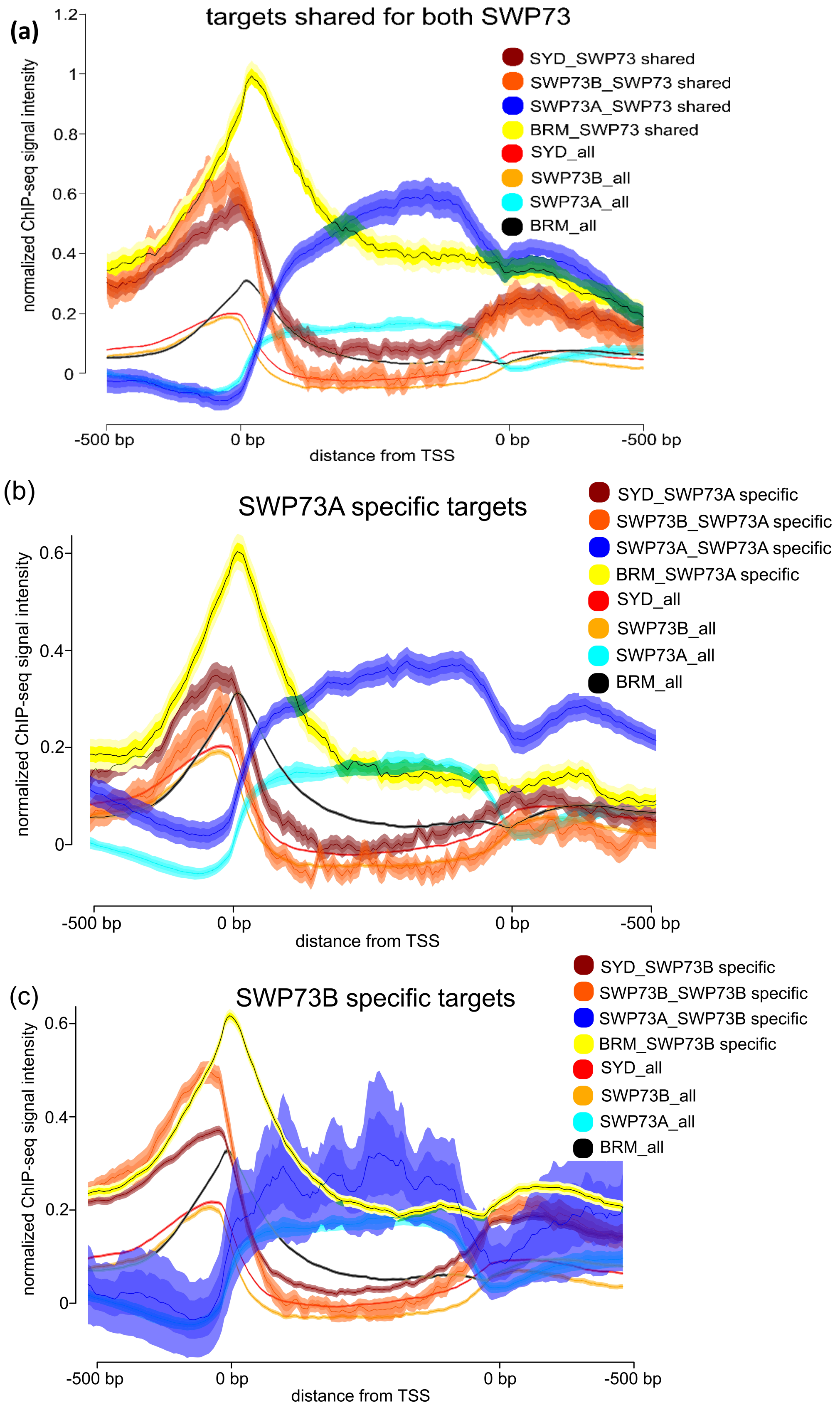

Fig. S3 The analysis of SWP73A, SWP73B, SYD, and BRM occupancy at specific and co-occupied gene sets in Arabidopsis. (a) Plots showing the occupancy of SWP73A, SWP73B, SYD, and BRM at genes co-occupied by SWP73A and SWP73B, as well as **(b)** genes uniquely occupied by either SWP73A (SWP73A-

spec) or **(c)** SWP73B (SWP73B-spec). Plot to all means all protein-coding genes from Arabidopsis (TAIR10).

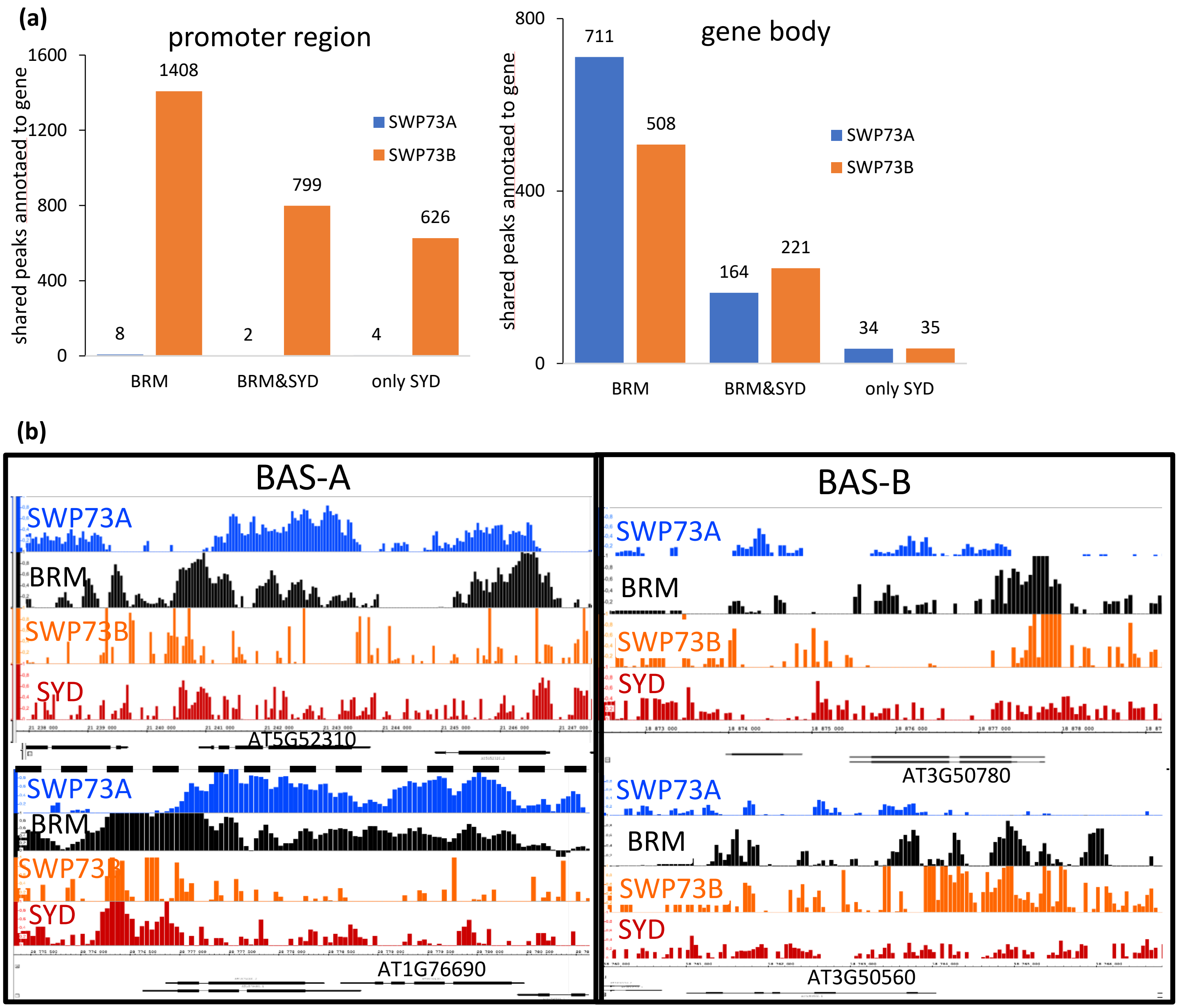

Fig. S4 SWP73A/SWP73B co-targeted genes and chromatin landscapes of BAS-A, BAS-B, and SAS. (a) Peaks annotated to genes specifically targeted by SWP73A (Huang *et al.*, 2021) and SWP73B (Jégu *et al.*, 2017), along with one or both ATPases—SYD (Shu *et al.,* 2021) and/or BRM (Liu *et al.*, 2016). **(b)** Examples of ChIP-seq tracks at representative *loci* illustrating targets specific to BAS-A, BAS-B, or shared among BAS-A, BAS-B, and SAS, or between BAS-B and SAS.

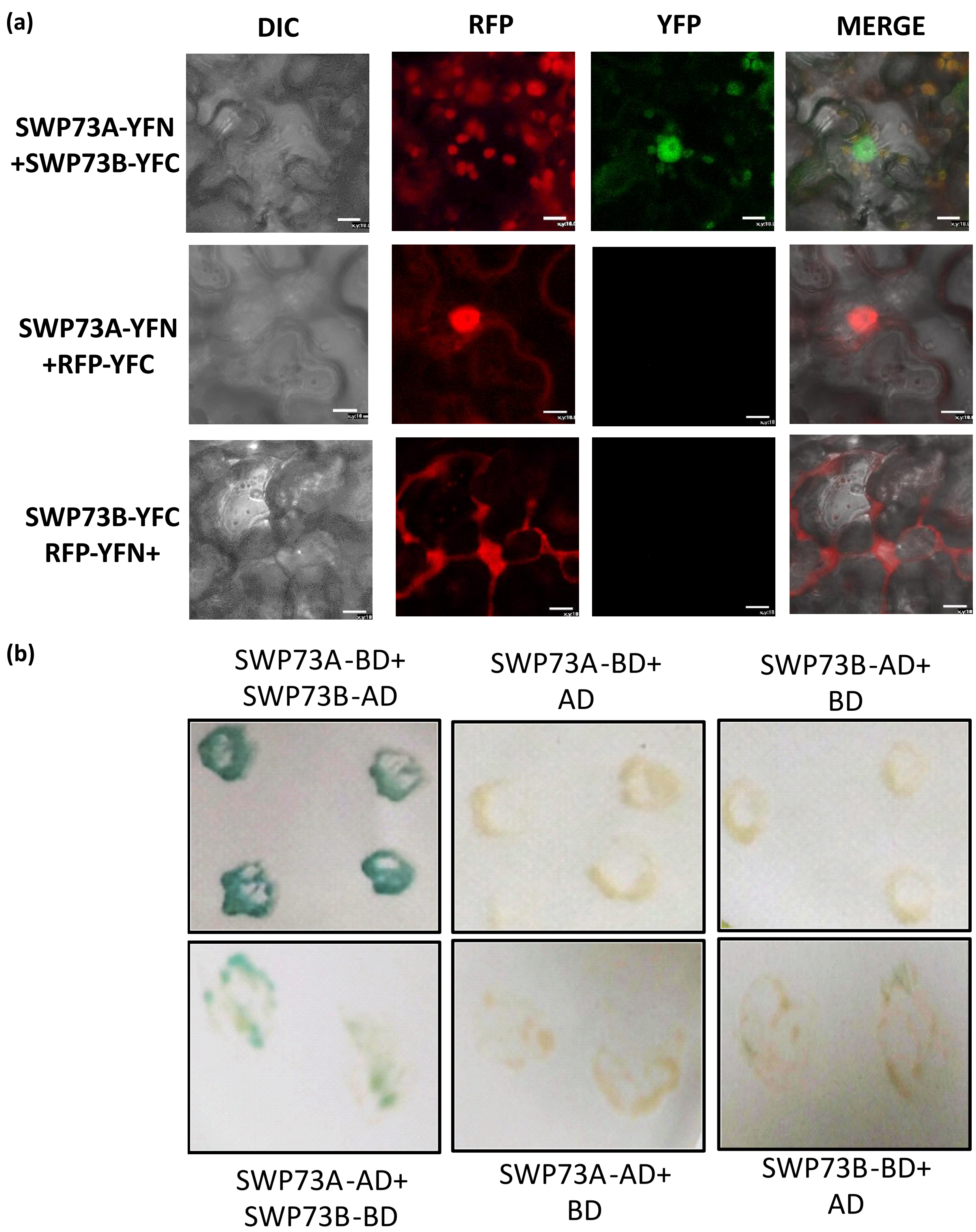

Fig. S5 Arabidopsis SWP73A and SWP73B directly interact. (a) BiFC assays of in vivo interactions of SWP73A and SWP73B proteins in wild tobacco (*Nicotiana benthamiana*) leaves. Left panel: YFP/RFP channel; right panel: differential interference contrast image. Negative controls for BiFC analysis include the RFP fused with the YFP-C-terminal or YFP-N-terminal domain. Bar = 10 µm. **(b)** Yeast two-hybrid assays showing SWP73A-SWP73B protein interactions (blue colour). Negative controls for the

yeast two-hybrid protein interaction assays were included. AD means active domain, BD – binding domain.

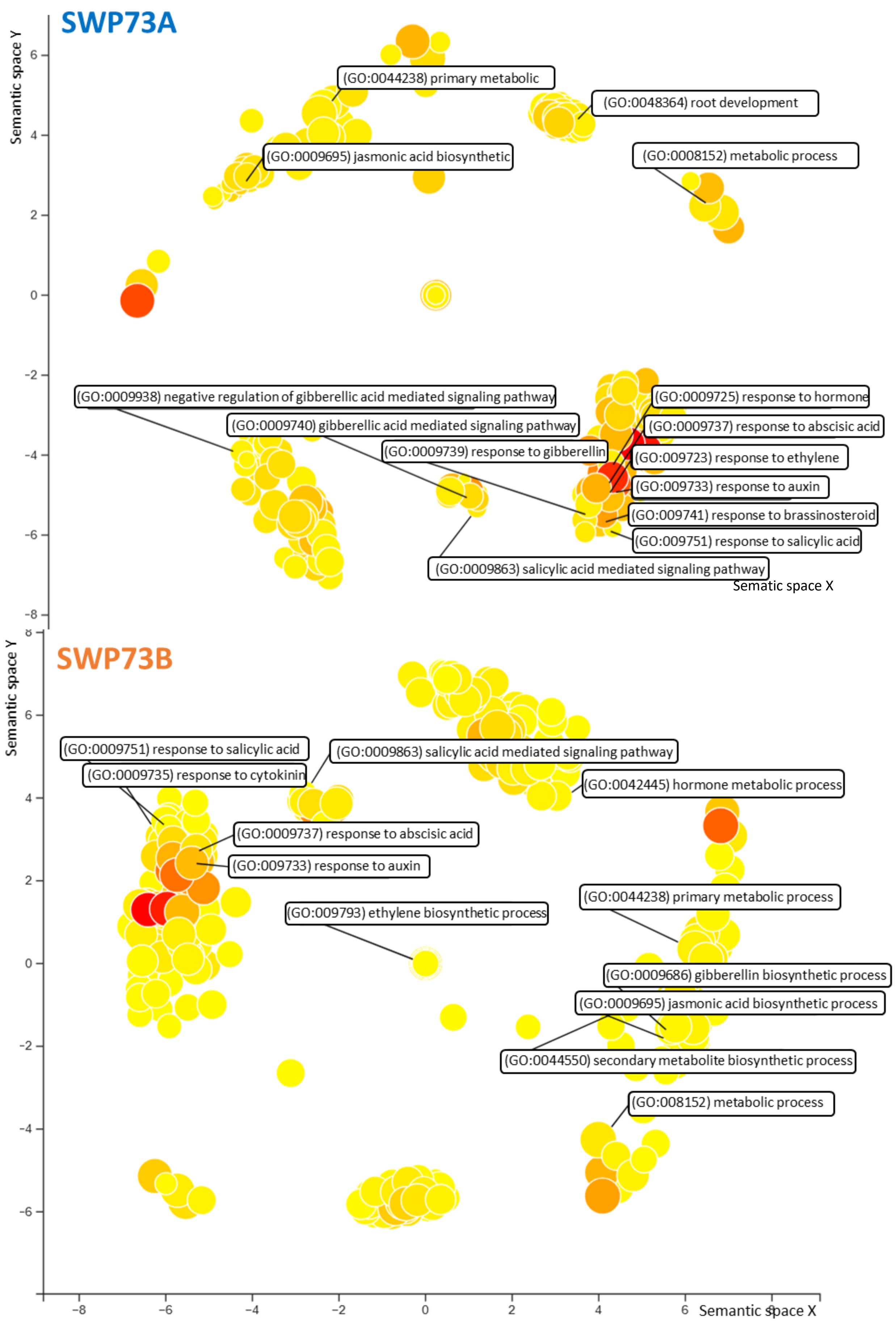
SWP73A
Semantic space Y
(GO:0044238) primary metabolic
(GO:0048364) root development
(GO:0009695) jasmonic acid biosynthetic
(GO:0008152) metabolic process
(GO:0009938) negative regulation of gibberellic acid mediated signaling pathway
(GO:0009725) response to hormone
(GO:0009740) gibberellic acid mediated signaling pathway
(GO:0009737) response to abscisic acid
(GO:0009739) response to gibberellin
(GO:009723) response to ethylene
(GO:009733) response to auxin
(GO:009741) response to brassinosteroid
(GO:009751) response to salicylic acid
(GO:0009863) salicylic acid mediated signaling pathway
Sematic space X
SWP73B
Semantic space Y
(GO:0009751) response to salicylic acid
(GO:0009735) response to cytokinin
(GO:0009863) salicylic acid mediated signaling pathway
(GO:0042445) hormone metabolic process
(GO:0009737) response to abscisic acid
(GO:009733) response to auxin
(GO:009793) ethylene biosynthetic process
(GO:0044238) primary metabolic process
(GO:0009686) gibberellin biosynthetic process
(GO:0009695) jasmonic acid biosynthetic process
(GO:0044550) secondary metabolite biosynthetic process
(GO:008152) metabolic process
Semantic space X

Fig. S6 SWP73A and SWP73B bind to genes involved in multiple hormonal pathways in Arabidopsis. Selected gene ontology classes of genes occupied by SWP73A **(a)** or SWP73B **(b)** and annotated to hormones pathways according to Arabidopsis Hormone Database (Jiang *et al.*, 2011).

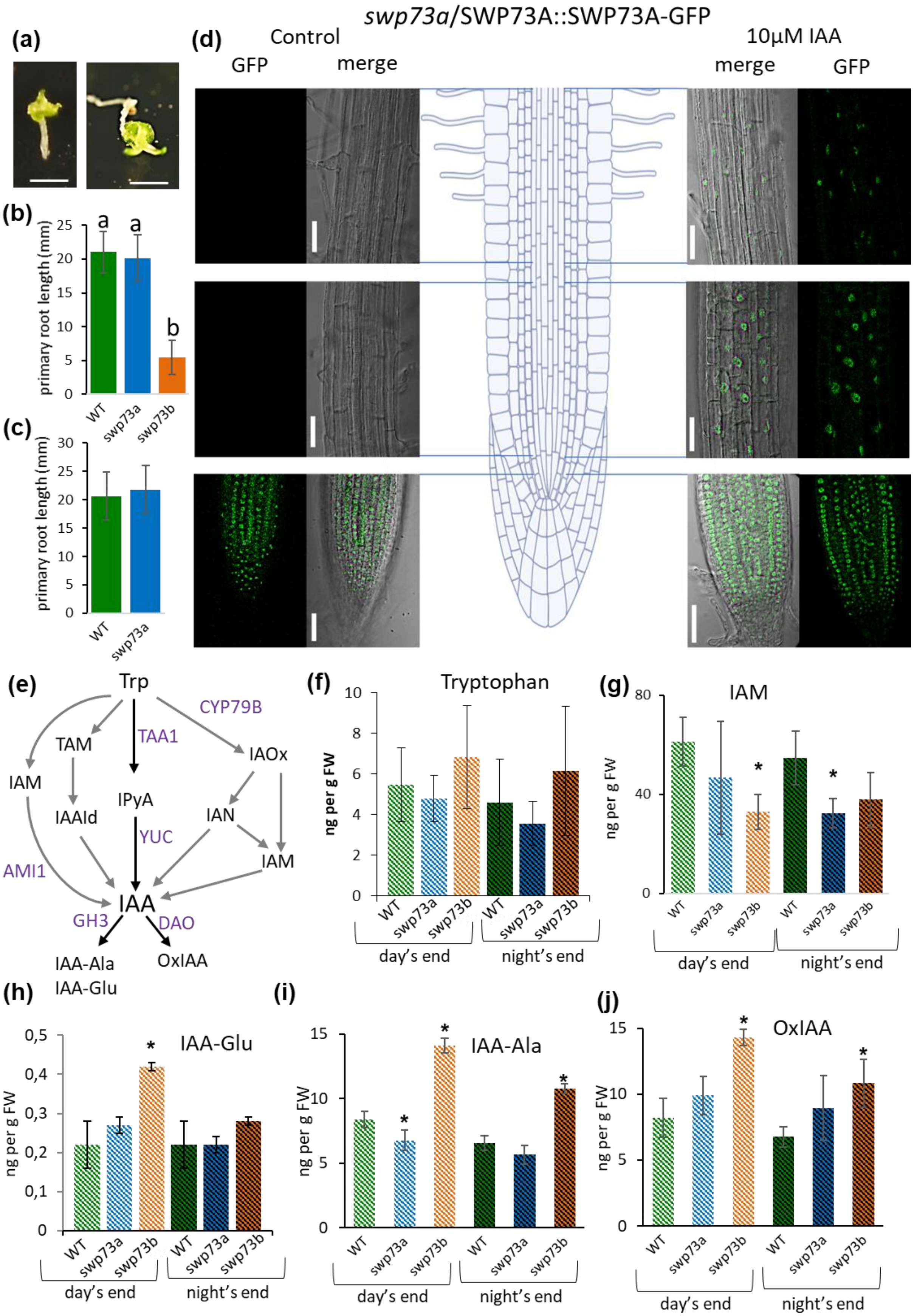

swp73a/SWP73A::SWP73A-GFP
(a)
(b)
primary root length (mm)
WT
swp73a
swp73b
(c)
(d)
Control
GFP
merge
10μM IAA
(e)
Trp
TAM
TAA1
CYP79B
IAOx
IAM
IPyA
IAAld
IAN
YUC
AMI1
IAA
GH3
DAO
IAA-Ala
IAA-Glu
OxIAA
(f)
Tryptophan
ng per g FW
day's end
night's end
(g)
IAM
(h)
IAA-Glu
(i)
IAA-Ala
(j)
OxIAA

Fig. S7 *swp73a* and *swp73b* Arabidopsis mutants features related to auxin biosynthesis and response. **(a)** The phenotype of *swp73b* plants grown on ½ MS supplemented with 10µM IAA. Scale bar: 1mm **(b)** Root length of WT, *swp73a* and *swp73b* measured 6 days after stratification. Lowercase letters show significant differences between genetic backgrounds, as determined by the ANOVA with *post hoc* Tukey HSD test. n> 50. **(c)** Root length of 10-day-old WT and *swp73a* plants after 4 days of treatment with 2 µM IAA. There is no difference between genetic backgrounds, as determined by the ANOVA with *post hoc* Tukey HSD test, n>35. **(d)** Localisation of SWP73A-GFP under native promoter after mock and 4h IAA treatment. Scale bar: 50µm. **(e)** Simplified scheme of auxin biosynthesis pathway in Arabidopsis based on (Mashiguchi *et al.*, 2011) and (Cao *et al.*, 2019). Auxin metabolites are marked in black: IAA (indole-3-acetic acid), IAM (indole-3-acetamide), IAN (indole-3-acetonitrile), IAGlu (indole-3-acetyl-L-glutamic acid), IAAla (indole-3-acetyl-L-alanine), OxIAA (2-oxindole-3-acetic acid). Enzymes are marked in purple. Black arrows indicate the main pathway, while grey arrows indicate alternative pathways. **(f)** Tryptophan, **(g)** IAM, **(h)** IAA-Glu, **(i)** IAA-Ala, and **(j)** OxIAA levels in WT, *swp73a* and *swp73b* mutants. Samples were collected shortly before night's or day's end from 3-week-old plants.

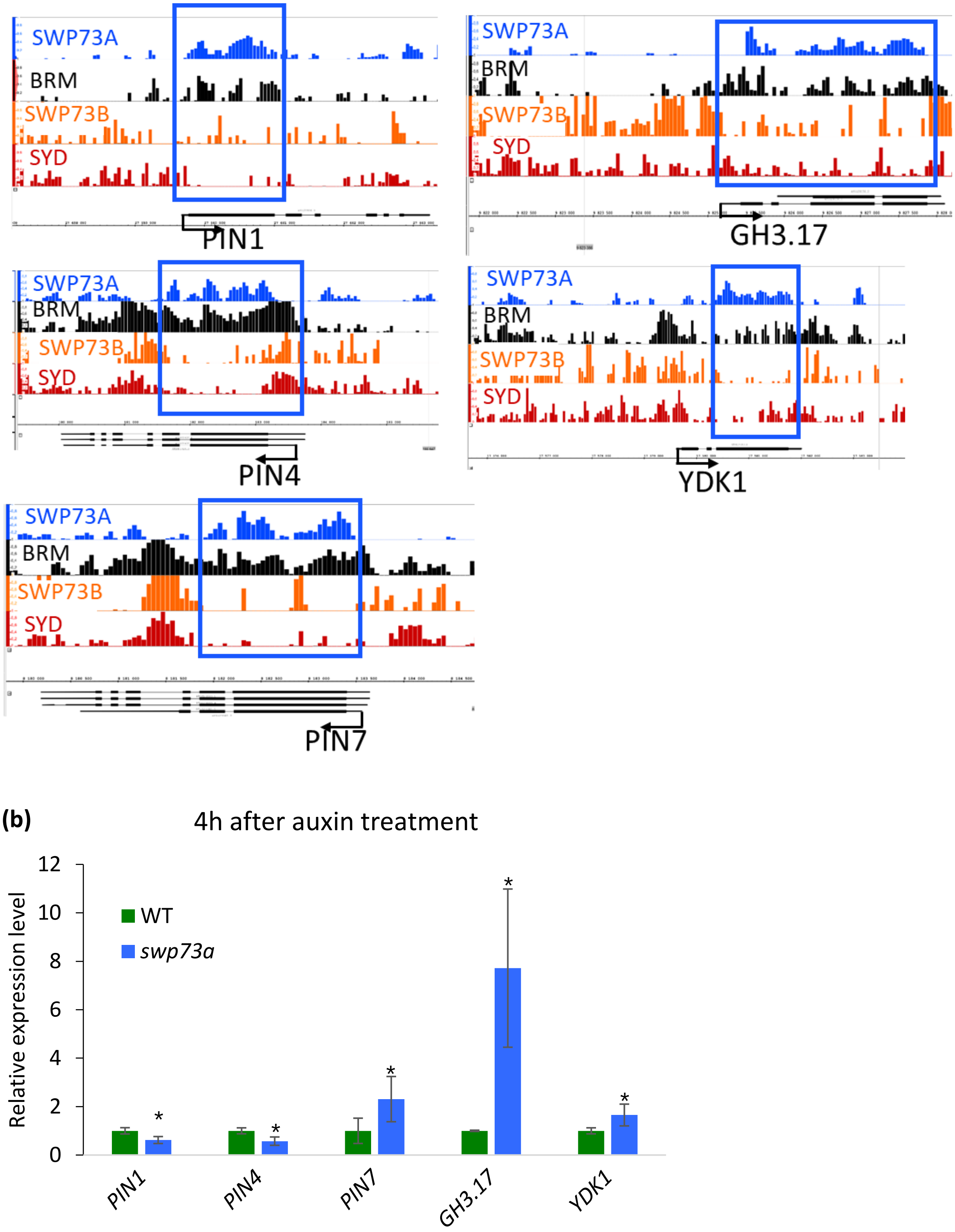

Fig. S8 Auxin-induced expression and ChIP-seq analysis of BAS-A Targets in Arabidopsis WT and *swp73a*. (a) Examples of ChIP-seq tracks at representative *loci*, corresponding to RT-qPCR, illustrating targets specific for BAS-A subcomplex containing SWP73A subunit. **(b)** The relative expression level of *PIN1*, *PIN2*, *PIN4*, *PIN7*, *GH3.17* and *YDK1* in WT and *swp73a* measured after spraying with 10 µM auxin.

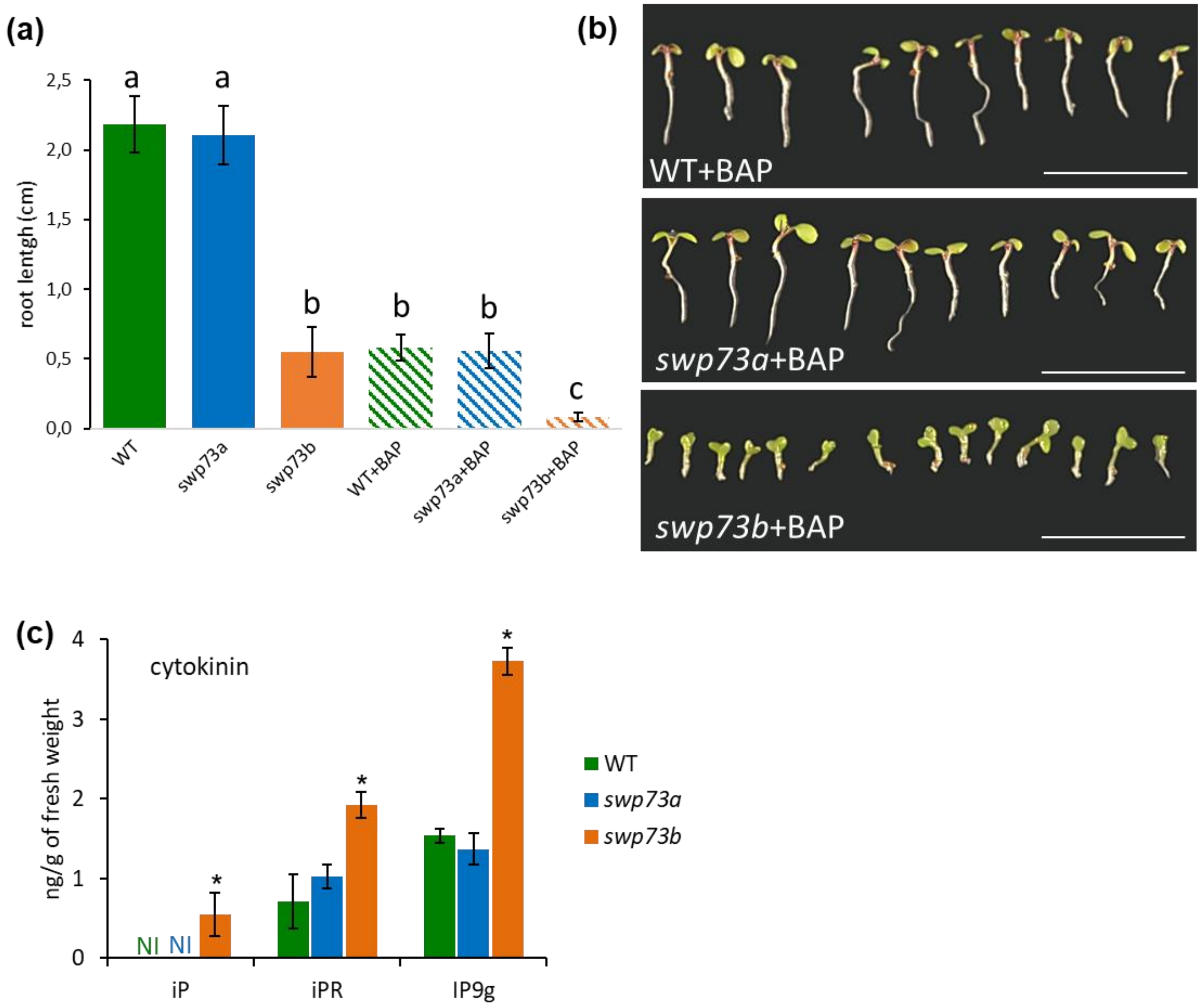

Fig. S9 BAP treatment and IP profiling in WT, *swp73a*, and *swp73b* indicate differential roles of Arabidopsis SWP73A and SWP73B in cytokinin signalling. (a) The effect of the treatment of *swp73a* and *swp73b* plants with 1µM BAP. Lowercase letters indicate significant differences between genetic backgrounds, as determined by the *Kruskal-Wallis ANOVA* with *post hoc* Dunn's test. **(b)** Cytokinin's hypersensitivity of *swp73b*. *swp73b* fails to accumulate anthocyanins in response to 1 µM BAP on ½ MS medium. (**c**) iP-Type cytokinins in *swp73a* and *swp73b.* N^6-(Δ^2-isopentenyl)adenine (iP), isopentenyladenine riboside (iPR), and iP N9-glucoside (iP9G) quantity is affected in *swp73b* mutant. NI means Not Identified.

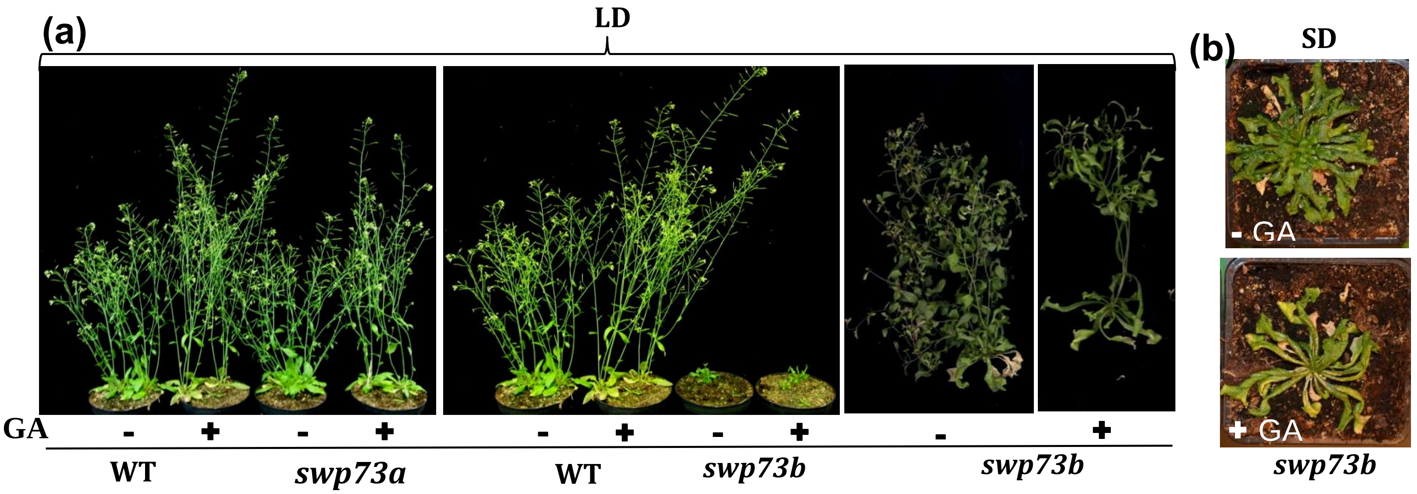

Fig. S10 Gibberellin treatment partially reverses *swp73b* developmental defects. Phenotype analysis of WT, *swp73a,* and *swp73b* growing under long-day (LD) or short-day (SD) conditions, sprayed with GA twice a week through the life cycle.

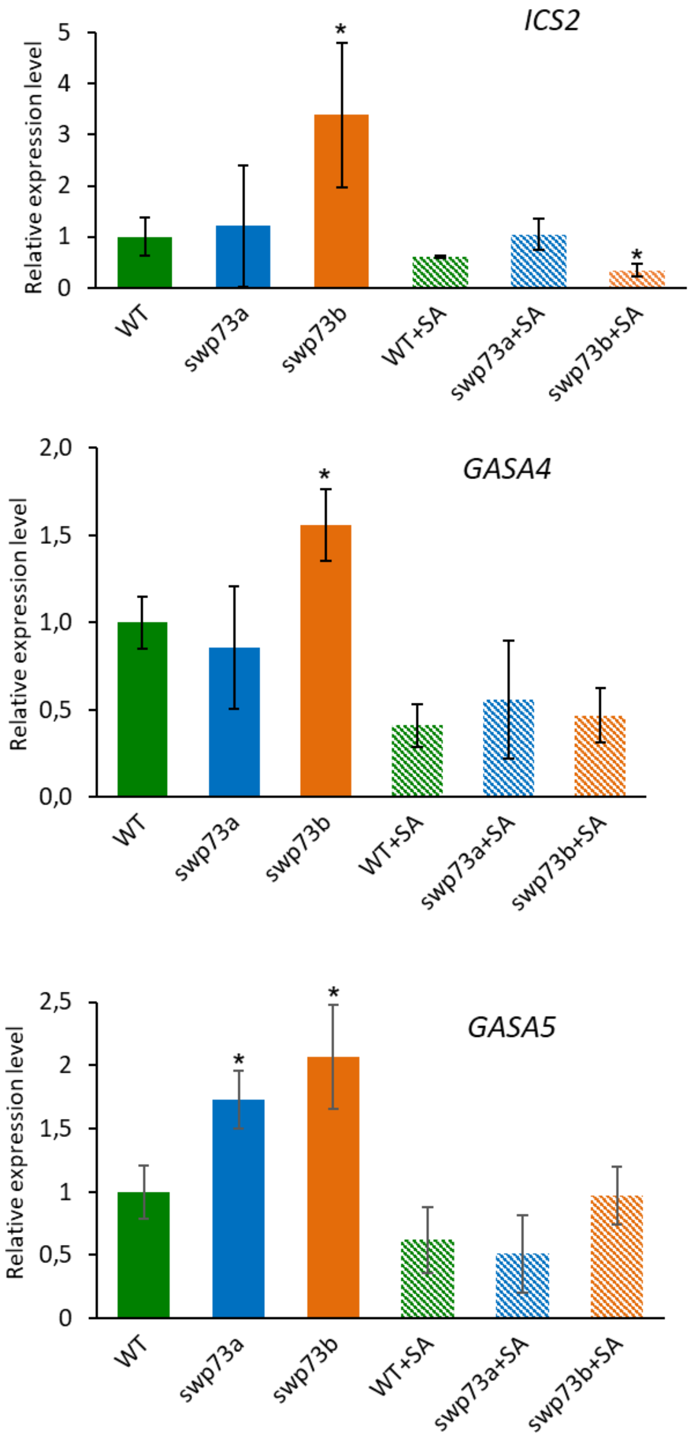

Fig. S11 Salicylic acid signal transduction is affected in *swp73* Arabidopsis mutant lines. The relative expression level of *ICS2*, *GASA4,* and *GASA5*. Values are average ± standard deviation obtained from three independent replicates. The asterisks denote statistically significant enrichment compared to the WT plant (*t*-test, *p*-value <0.05).

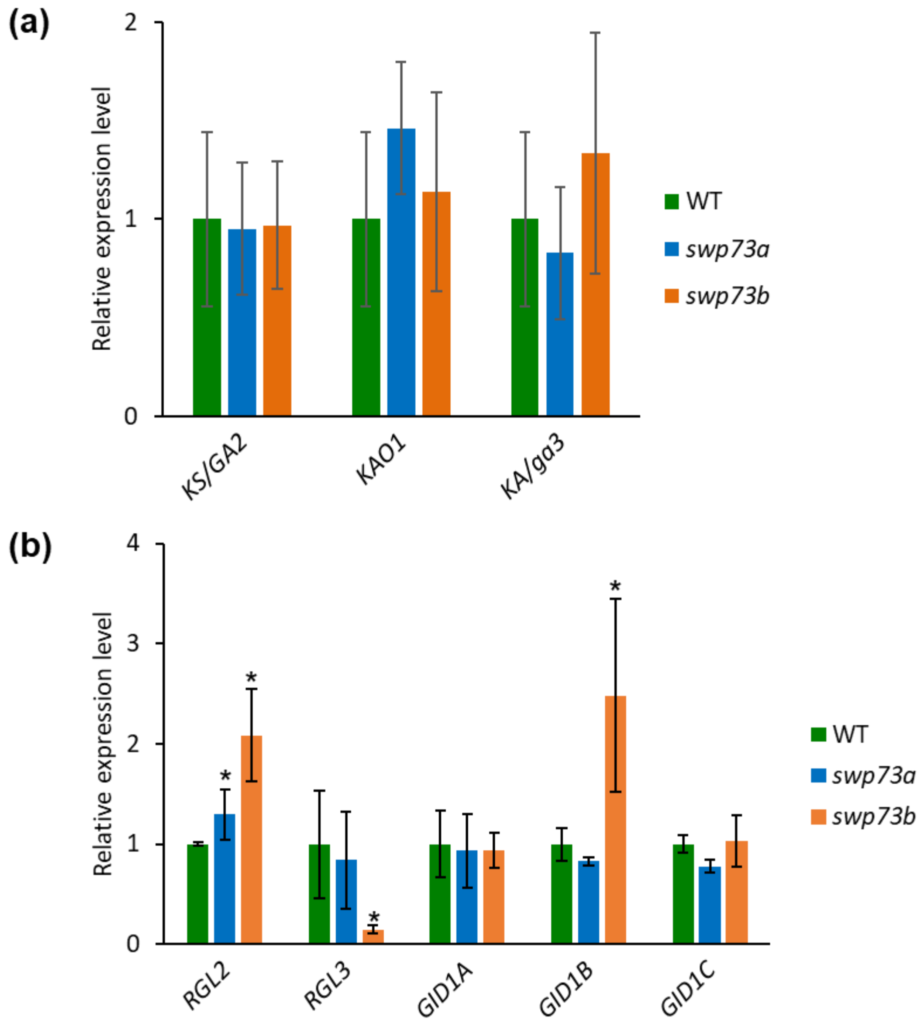

Fig. S12 SWP73 do not control the expression of genes involved in the initial steps of gibberellin biosynthesis in Arabidopsis. (a) Unaffected expression of genes encoding enzymes involved in the first steps of GA biosynthesis. Values are average ± standard deviation obtained from three independent replicates. **(b)** The relative expression level of *RGL2*, *RGL3* - GA repressors, and *GID1A*/*B/C* - GA receptors *inswp73a* and *swp73b*.

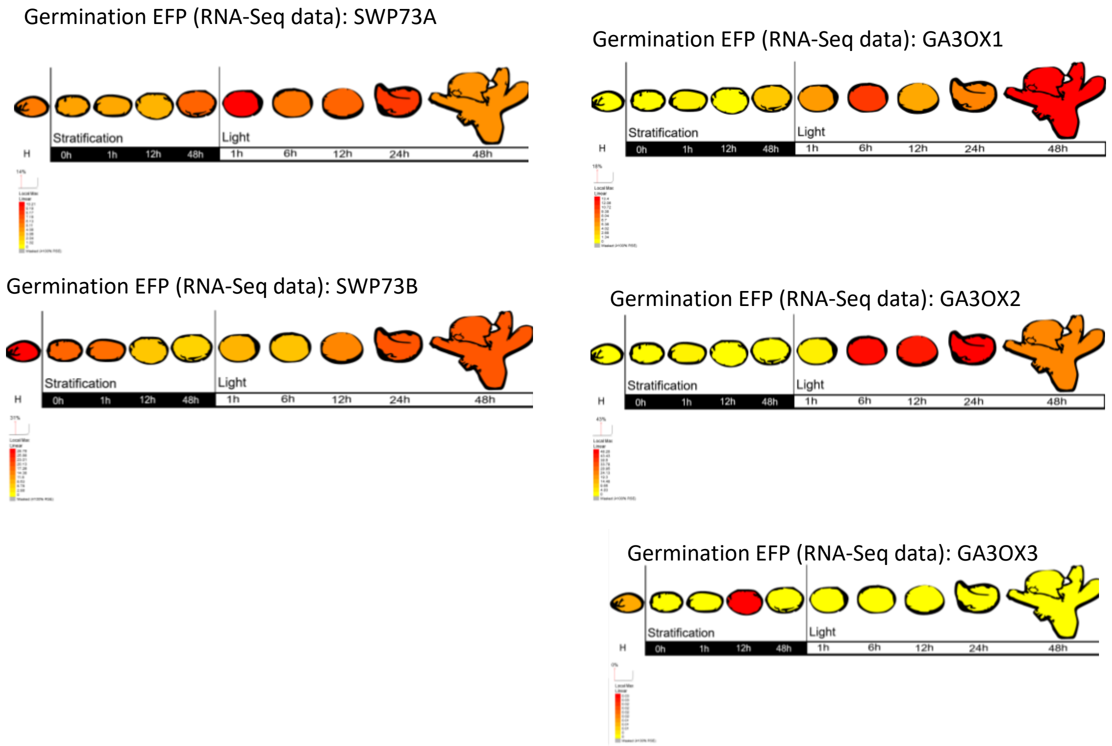

Fig. S13 Expression patterns of Arabidopsis *SWP73A, SWP73B, GA3OX1, GA3OX2* and *GA3OX3* genes presented in EFP public databases during germination.

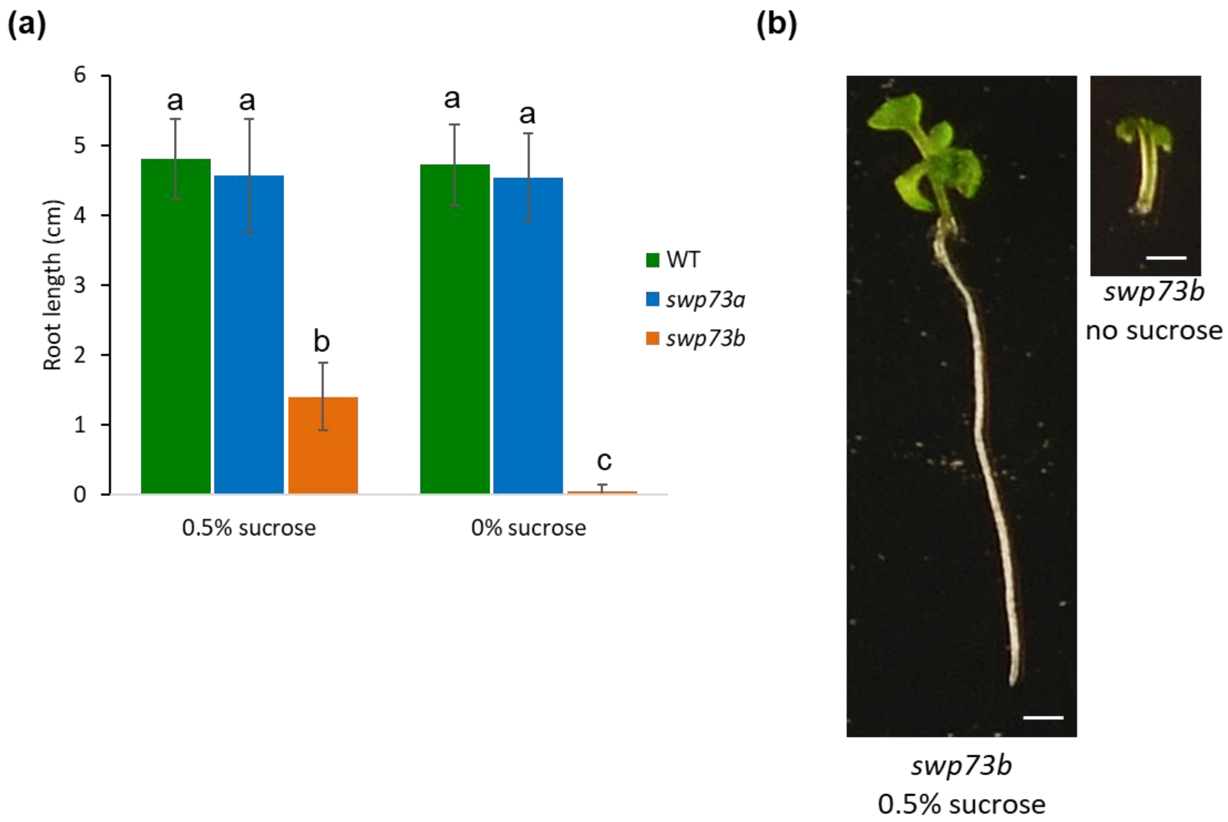

Fig. S14 *swp73a* and *swp73b* exhibit differential Arabidopsis response to the presence of sucrose in the medium. (a) Root length of WT, *swp73a,* and *swp73b* mutants growing on medium with or without sucrose. Letters correspond to statistical significance ($P < 0.001$) as determined by the *Kruskal-Wallis ANOVA* with *post hoc* Dunn's test. **(b)** Representative picture of *swp73b* growing on medium with or without sucrose. The bar indicates 1cm.

References

Cao X, Yang H, Shang C, Ma S, Liu L, Cheng J. 2019. The Roles of Auxin Biosynthesis YUCCA Gene Family in Plants. *International Journal of Molecular Sciences* 20: 6343.

Mashiguchi K, Tanaka K, Sakai T, Sugawara S, Kawaide H, Natsume M, Hanada A, Yaeno T, Shirasu K, Yao H, *et al.* 2011. The main auxin biosynthesis pathway in *Arabidopsis*. *Proceedings of the National Academy of Sciences* 108: 18512–18517.

Huang C-Y, Rangel DS, Qin X, Bui C, Li R, Jia Z, Cui X, Jin H. 2021. The chromatin-remodeling protein BAF60/SWP73A regulates the plant immune receptor NLRs. *Cell Host & Microbe* 29: 425-434.e4.

Jégu T, Veluchamy A, Ramirez-Prado JS, Rizzi-Paillet C, Perez M, Lhomme A, Latrasse D, Coleno E, Vicaire S, Legras S, *et al.* 2017. The Arabidopsis SWI/SNF protein BAF60 mediates seedling growth control by modulating DNA accessibility. *Genome Biology* 18: 114.

Jiang Z, Liu X, Peng Z, Wan Y, Ji Y, He W, Wan W, Luo J, Guo H. 2011. AHD2.0: an update version of Arabidopsis Hormone Database for plant systematic studies. *Nucleic Acids Research* 39: D1123–D1129.

Li C, Gu L, Gao L, Chen C, Wei CQ, Qiu Q, Chien CW, Wang S, Jiang L, Ai LF, Chen CY, Yang S, Nguyen V, Qi Y, Snyder MP, Burlingame AL, Kohalmi SE, Huang S, Cao X, Wang ZY, Wu K, Chen X, Cui Y. 2016. Concerted genomic targeting of H3K27 demethylase REF6 and chromatin-remodeling ATPase BRM in Arabidopsis. *Nat Genet.* 48:687-693.

Shu J, Chen C, Li C, Thapa RK, Song J, Xie X, Nguyen V, Bian S, Liu J, Kohalmi SE, Cui, Y. 2021. Genome-wide occupancy of Arabidopsis SWI/SNF chromatin remodeler SPLAYED provides insights into its interplay with its close homolog BRAHMA and Polycomb proteins *Plant J*, 106: 200-213
